# Better together: Group-level social dynamics predict individuals’ psychological adjustment during a major life transition

**DOI:** 10.1177/02654075251401692

**Published:** 2025-11-24

**Authors:** Marisa A. Nelson, Julia S. Nakamura, Frances S. Chen, Amori Yee Mikami

**Affiliations:** 1 8166 University of British Columbia, Canada

**Keywords:** Emerging adulthood, psychological well-being, friendship, depression, belongingness

## Abstract

Establishing new social connections is critical during the transition to university, especially for students who have moved far from home. We examined how students’ one-on-one connections, along with the dynamics of their social groups, related to their adjustment across a school year. Participants were 717 first-year international undergraduates at a Canadian university who participated in a transition program involving assignment to orientation groups of 20-30 students. Cross-sectionally, students’ individual-level social network measures were associated with better adjustment at the start of the year. Longitudinally, being part of a group with better social connections at the start of the year was associated with students’ higher grade point average, lower hostility, and greater institutional attachment at the end of the school year. Findings highlight the importance of group-level social dynamics for emerging adults’ adjustment during a significant life transition.

Social connection is a fundamental human need. Among adolescents and young adults, social connectedness has been associated with higher confidence, hope, and life satisfaction (Jakobsen et al., 2022; Jose et al., 2012), as well as greater meaning in life (Blau et al., 2016; Jakobsen et al., 2022). Conversely, a lack of social connection (e.g., loneliness, social isolation) during adolescence and young adulthood has been associated with depression and anxiety (McIntyre et al., 2018), as well as poorer sleep quality, health behaviors, and physical health (Christiansen et al., 2021; Matthews et al., 2017; Simone et al., 2022).

## Importance of the individual level and the group level

Much of the existing work on this topic has taken an individual-level perspective, focusing on how the one-on-one connections that someone has, or lacks, relate to their adjustment as an individual, without accounting for the social context. Yet, an individual’s relationships with others are almost always nested within broader social groups. Imagine someone has just joined a new group setting, such as a gym or a club, and they arrive for the first time in an unfamiliar space with unfamiliar people. The group is lively, and they are greeted by a scene full of laughter, animated conversation, and connection, drawing them into the room. The warm glow of this interaction may promote their sense of belonging independent of their individual-level interactions or influence their subsequent individual-level interactions in that setting. As shown in this example, the dynamics and culture of social groups are shaped by, and can also affect, all individuals within them, making it important to consider the social network in which someone’s relationships are situated (Bronfenbrenner & Ceci, 1994). In the current study, we sought to understand not only individual-level relationships (e.g., one’s own social connections), but also group-level social dynamics (e.g., the networks that define the broader social milieu in which one’s social connections are situated), that may be associated with individuals’ adjustment.

While prior psychological research has shown myriad individual-level effects of social connection on adjustment, growing evidence suggests that the group social environment also matters. For example, among elementary and secondary school students, the teacher’s practices (at the classroom level) can shape an emotionally supportive classroom climate. This classroom climate, in turn, predicts students’ subsequent individual emotional, behavioral, and academic adjustment (Aldrup et al., 2018), social development (Mikami et al., 2012), and academic achievement (Hamre & Pianta, 2005). As another example, the overall level of cohesiveness between members in a therapy group predicts individual-level treatment outcomes for the individuals in that group (Burlingame et al., 2018).

Related work in sociology and public health also emphasizes the importance of social group dynamics on individual-level outcomes. For instance, Fowler and Christakis (2008) found that an individuals’ happiness relates to the happiness of others in their social network, even those with whom they do not directly interact. In some cases, group-level effects are seen where individual-level effects are not. For example, the performance of individuals in a sales team was better explained by their social network structure than by their individual characteristics (Carboni & Ehrlich, 2013).

These findings suggesting the power of the group social environment are consistent with social identity theory, which posits that people develop shared social identities within the groups to which they belong (e.g., family, friends, cultural groups), and that identification with such groups can influence individuals’ attitudes and behaviors (Tajfel & Turner, 1986). Groups carry important social norms, or rules for how individuals should behave (Smith & Louis, 2009); norms tend to be highly influential when someone strongly identifies with the group (Schofffild et al., 2001; Terry & Hogg, 1996), and when the norms come from important reference groups like friends (Kwon & Lease, 2009; Zhou et al., 2023). Thus, broad networks of social relationships, and the norms within networks, can exert strong influences on individuals’ attitudes and behaviors.

## Social connections and major life transitions

Social connections play an important role during major life transitions, such as moving away from one’s childhood home or starting one’s first job. Life transitions are often stressful and can negatively impact individuals’ adjustment, even if the transition is viewed as positive (e.g., getting married; Iyer et al., 2009; Moustafa et al., 2020). Social connection may buffer against these risks. For example, both young adults and older adults undergoing a life transition were less likely to report being depressed if they developed new social ties (Gillath et al., 2011).

Beginning university is a major life transition for young adults, during which they are at heightened risk for developing depression and anxiety (Kroshus et al., 2021), and hostility (Galambos & Krahn, 2008). International students, who are living away from their family and cultural groups, face some unique challenges during the transition to university. Homesickness, culture shock, language barriers, and perceived discrimination (Oduwaye et al., 2023) may all exacerbate international students’ risks for maladjustment and feelings of not belonging. Indeed, international students report high levels of loneliness and disconnection—with one qualitative study of over 200 participants finding that two-thirds of international students experienced loneliness (Sawir et al., 2008). As such, social connection may be an important buffer against maladjustment for international students.

Group-level social connections may be especially influential during transitions, which are often accompanied by co-occurring shifts in group membership (Iyer et al., 2009). Notably, the transition to university is a time when social networks undergo major changes, and social groups rapidly form and evolve. Because they have moved away from social support systems in their home country, identification with friend groups at university may have a strong influence on international students’ attitudes and behaviors, in line with social identity theory. Thus, the transition to university represents a unique opportunity for studying the relationships between group-level connections and adjustment, and these processes may be particularly salient for international students.

## Current study

We assessed how individual-level social connections and group-level social dynamics relate to individuals’ adjustment over the course of an academic year among a sample undergoing a major life transition: first-year international undergraduate students who have moved away from their homes and local support systems to attend a Canadian university. These students were participating in a transition program where they were nested in orientation groups called “learning communities.” We used social network analysis to assess relationships between individuals along with the group social context in which individuals’ relationships are situated (Lusher et al., 2010). To examine individual-level social connections, we assessed individuals’ reciprocated friendships and considered four measures of individuals’ network centrality with the other members of their learning community (see Table 1 for information on the social network measures used). To examine group-level social dynamics, we evaluated the learning community mean of social network measures (i.e., the overall pattern of centrality and reciprocated friendships in the learning community as a whole) to understand the larger social context in which individuals were situated, above and beyond their individual-level social connections.

**Table 1.** Social network measures.

Measure	Description
Network centrality	
In-degree	Total number of incoming friendship nominations received by an individual. This measure represents how well-liked an individual is in their network
Out-degree	Total number of friendship nominations sent out by an individual. This measure represents how well-connected an individual perceives themselves to be in their network or their self-perceived social acceptance
In-eigenvector	Total number of incoming friendship nominations an individual received, weighted by the importance or connectedness of the person sending the friendship tie. More connected friends contribute more to an individual’s in-eigenvector centrality than less connected friends. This measure represents how well-liked an individual is while taking into consideration how well-liked the people who nominated them are
Out-eigenvector	Total number of outgoing friendship nominations an individual sent, weighted by the importance or connectedness of the person receiving the friendship tie. More connected friends contribute more to an individual’s out-eigenvector centrality than less connected friends. This measures how well-connected an individual perceives themselves to be while taking into consideration how well-connected the people they nominated are
Reciprocated friendship ties	Total number of outgoing friendship nominations an individual sent that were reciprocated by the recipient; meaning, both individuals in the dyad needed to nominate one another as friends. This represents a mutual connection which can be considered to reflect a stronger friendship tie

We considered five key aspects of adjustment in our sample. Specifically, we examined individuals’ depression, anxiety, and hostility, all of which can be elevated in university students (Ibrahim et al., 2013) and exacerbated by the social upheaval that often accompanies starting university (Hamaideh, 2011). We also examined individuals’ grade-point averages (GPAs) and sense of belonging at their university, as these are important markers of students’ academic adjustment and predictors of retention (Luo et al., 2020; Pedler et al., 2022).

Our first aim was to examine cross-sectional associations between individual- and group-level social network measures and adjustment, at the start of the academic year. We hypothesized that individuals who had higher individual-level network centrality or reciprocated friendships in their learning community (reflecting their one-on-one social connections with other people) would have better concurrent adjustment (i.e., higher GPAs and sense of belonging; lower anxiety, depression, and hostility). At the group level, we hypothesized that individuals in learning communities with higher group-level centrality or reciprocated friendships (reflecting the overall social dynamics of the group) would have better concurrent adjustment.

Our second aim was to test longitudinal relationships between social network measures at the start of the academic year (baseline) and individuals’ adjustment at the end of the academic year (follow-up), while statistically adjusting for start of year adjustment. Here, we hypothesized that higher individual-level network centrality and reciprocated friendships would each be associated with individuals showing better academic and psychological adjustment at the end of the year. At the group level, we hypothesized that higher learning community-level group means of these measures at the start of the year, would each be associated with individuals showing better academic and psychological adjustment at the end of the year.

## Method

### Participants

Study participants were 717 incoming first-year undergraduate students (*M* age = 18.70; 58.7% women, 41.3% men) at a large public Canadian university. We combined data collected from two cohorts of students from the 2012-2013 and 2013-2014 academic years. The participants were international students (most of whom attended high school outside of Canada) and were enrolled in “Jump Start,” a transition program developed to facilitate their adjustment to university. Table 2 provides demographic information for the sample, including the country in which they completed high school prior to transitioning to university in Canada.

**Table 2.** Characteristics of study participants (*N* = 717).

Variable	*M* (SD)	*N* (%)
Age (years); range 16.7–32.3	18.70 (0.96)	
Gender		
Women (%)		421 (58.7)
Men (%)		296 (41.3)
Family income (in CAD; median)	$93,204	
Country of high school^a,b^		
United States		191 (26.8)
China		131 (18.4)
Canada^c^		40 (5.6)
Other Asia		208 (29.2)
Europe		54 (7.6)
South America		32 (4.5)
Africa		30 (4.2)
Other North America		23 (3.2)
Oceania		3 (0.4)
Baseline measures		
High school GPA	83.46 (8.90)	
Fall depressive symptoms	1.94 (0.81)	
Fall anxiety symptoms	1.76 (0.71)	
Fall hostility	1.48 (0.59)	
Fall institutional attachment	7.16 (1.11)	
Follow-up measures		
University GPA	71.56 (11.12)	
Spring depressive symptoms	2.01 (0.90)	
Spring anxiety symptoms	1.76 (0.81)	
Spring hostility	1.57 (0.68)	
Spring institutional attachment	7.04 (1.21)	

^a^Individuals were eligible to participate in Jump Start if either (a) they were an international student with a home country outside of Canada, or (b) they were legally a Canadian citizen or permanent resident, but completed high school outside of Canada. Here, we report where students completed high school as this represents where they lived most recently prior to their arrival on campus for Jump Start.

^b^Percentages may not add up to 100% due to rounding.

^c^Thirty two of the participants in this group were international students, but completed high school in Canada. The other seven participants were Canadian citizens and attended high school in Canada, but were eligible for Jump Start because they self-identified as Indigenous.

Jump Start attendees arrived on campus 2 weeks prior to the start of the fall semester, where they were assigned to learning communities, which were smaller orientation groups of around 20-30 students, based on their broad academic interests. The 717 participants in our sample were nested in 59 learning communities. To our knowledge, all members within a learning community were previously unacquainted. Jump Start was explicitly designed to provide support for new international students in three areas: (a) practical, such as opening a Canadian bank account; (b) academic, such as understanding the norms and expectations of university in Canada; and (c) social, such as offering opportunities to build positive social connections. During the 2-week Jump Start program, learning communities spent approximately 8 hours each day together in structured activities and lived on campus on the same dormitory floor. Activities included social and recreational events, daily group meetings with faculty, and workshops. Although structured activities ended at the conclusion of the 2-week program, learning communities continued to live in the same on-campus dormitory, although they often moved to different floors. Nonetheless, living in the same dormitory provided opportunities to remain connected over the academic year. The unique nature of Jump Start offered an in-depth picture of social networks as they were being established during a major life transition.

### Study procedures

Study procedures were approved by the university’s research ethics board. Written consent was obtained from participants. The current investigation is a secondary data analysis of a portion of the data collected in the original study.

During the 2-week orientation program, all students enrolled in Jump Start were invited to participate in the study, and 1,226 of 1,688 eligible students consented to do so. Participants were asked to complete questionnaires approximately 2–3 weeks into the fall semester (i.e., shortly after the end of the Jump Start program); this was the baseline timepoint. This timing was chosen so that participants would have an opportunity to start classes and experience typical life at university. Participants received an individualized link to an online survey and were asked to complete it in private. Participants reported on their high school GPA, psychological adjustment, and attachment to their university. They also completed a sociometric measure used to create the social network variables at baseline, in which they were shown a list of the people in their learning communities and asked to indicate whether or not they considered each of their group members to be a friend.

Of the consented participants, 781 completed this sociometric measure. Social network analysis is particularly sensitive to missing data given the dependent nature of the relationships between variables (Smith & Moody, 2013). Using list-wise deletion, we removed 34 individuals with ≥20% of their social network data missing. Three additional individuals were omitted from analyses because their learning community had few participants (<4) who completed the baseline survey or consented for their data to be used. Finally, 27 participants from two learning communities were removed due to multiple individuals failing to identify themselves in the survey, meaning we could not attribute their social network data to their participant information. This resulted in a final sample of 717 participants nested in 59 learning communities.

At the end of the academic year, all participants completed another online survey in which they were asked the same questions about psychological adjustment and institutional attachment. We also obtained participants’ first-year university grades from the registrar. We considered this to be the follow-up timepoint. While participants had measures of GPA, psychological adjustment, and institutional attachment at both baseline and follow-up, we only analyzed the social network centrality and reciprocated friendship measures taken at baseline. While we did administer the sociometric measure at follow-up, these data are of questionable reliability, as many participants had trouble remembering who was in their original learning communities, or said they no longer interacted with these members. Although learning community members continued to live in the same dormitory building during the academic year, additional students moved into the building, and learning communities no longer resided on the same floor. Of the 717 participants who were in our baseline sample, 655 completed the follow-up survey.

### Measures

#### GPA

At baseline, participants self-reported their overall high school GPA. Based on the grading system in their home country, their average was converted to the Canadian percentage system (0–100%). At follow-up, participants’ first-year university grades were provided by the university registrar. To calculate a weighted GPA, each of their grades (expressed as a percentage of 100) was multiplied by the credit value for each course and the product was divided by the total number of credits completed. The university registrar also provided the number of credits attempted, which we used as a covariate in the models with GPA as an outcome variable.

#### Psychological adjustment

Participants completed the Depression, Hostility, and Anxiety subscales of the Brief Symptom Inventory (BSI) developed by Derogatis and Melisaratos (1983), at both baseline and follow-up. These three subscales were selected based on their relevance to a community sample of university students and given the high prevalence of psychological distress among this population (Galambos & Krahn, 2008; Heumann et al., 2024; Kroshus et al., 2021).

Depression subscale items were: thoughts of death or dying, feeling lonely, feeling blue, feeling no interest in things, feeling hopeless about the future, and feelings of worthlessness (Cronbach’s α = .85 [baseline]; .88 [follow-up]). Anxiety subscale items were: nervousness or shakiness inside, suddenly scared for no reason, feeling fearful, feeling tense or keyed up, spells of terror or panic, and feeling so restless that [they] couldn’t stay still (Cronbach’s α = .82 [baseline]; .87 [follow-up]). Hostility subscale items were: feeling easily annoyed or irritated; temper outbursts that [they] could not control; having urges to beat, injure, or harm someone; having urges to break or smash things; and getting into frequent arguments (Cronbach’s α = .77 [baseline]; .83 [follow-up]). Participants were asked to rate, for each item, how often over the past 7 days they were distressed by the symptom using a 5-point Likert scale ranging from 0 (*not at all*) to 4 (*extremely*). The mean of the items on each subscale was calculated, with higher scores indicating greater distress.

#### Institutional attachment

Participants completed the Institutional Attachment subscale of the Student Adaptation to College Questionnaire (Baker & Siryk, 1986), at baseline and follow-up. This subscale assessed students’ sense of belongingness at their university, using items such as, “I fit in well with the environment at (university name)” and “I am pleased with my decision to attend (university name).” At baseline, questions were framed to capture participants’ expectations (e.g., “I will fit in”) while at follow-up questions, were framed to capture their experiences (e.g., “I fit in”). Participants answered 15 questions using a Likert-scale ranging from 1 (*applies very closely to me*) to 9 (*doesn’t apply to me at all*). Negatively-worded items were reverse scored. Scores were averaged, with higher mean scores indicating greater attachment to their university (Cronbach’s α = .86 [baseline]; .86 [follow-up]).

#### Network centrality

At baseline, participants completed a sociometric survey that contained a list of the members of their learning community who had consented to the study. For each member, participants were asked to indicate whether they “considered this person to be a friend,” by adding a check next to that person’s name. These nominations were used to generate four measures of individuals’ centrality (see Table 1), using UCINET software. Centrality measures reflect the importance or connectivity of an individual within a social network. First, we considered in-degree centrality (how many people considered an individual as their friend) and out-degree centrality (how many people an individual listed as their friend). However, incoming and outgoing ties on their own may not sufficiently capture an individuals’ access to their network (e.g., Fowler & Christakis, 2008; Sandstrom & Dunn, 2014). Eigenvector centrality gives more weight to ties with well-connected others relative to ties with less-connected others. Individuals with greater eigenvector centrality may have more access to their networks and the resources that flow through them (Borgatti, 2005). We considered eigenvector centrality for both incoming ties (in-eigenvector) and outgoing ties (out-eigenvector). In all analyses, we adjusted for the number of participants in the learning community who completed the sociometric measure.

#### Reciprocated friendships

A friendship tie was considered reciprocated if both members of a dyad indicated the other as a friend on the sociometric survey (Table 1). Our analyses adjusted for the number of participants in the learning community who provided sociometric data.

#### Demographic covariates

In the baseline survey, participants reported their age (continuous), gender (0 = *man*; 1 = *woman*), and family income (converted to Canadian dollars; continuous).

### Data analytic plan

All analyses were conducted using R statistical software version 4.5.0. To accommodate the nested structure of the data, we used multilevel modeling (R lme 4 package v.1.1.37.1, Bates et al., 2015; R lmerTest package v.3.1.3, Kuznetsova et al., 2017) with random intercepts and fixed slopes. Participants were placed at level 1 and were nested in learning communities at level 2. All continuous variables at level 1 were group-mean centered, and income was standardized. In between-group analyses, unstandardized coefficients for the predictors in each model represent the expected change in the outcome variable for a one-unit increase in the predictor.

Tables 4 and 5 contain the intraclass correlation coefficients (ICCs) for each outcome variable. ICCs were low for many models (<5%), and for several of our outcome variables, the random effects intercept variance was 0. In this case, many would run a normal regression. Because group-level effects were of theoretical interest to us, and because the structure of the dataset was nested, we proceeded to conduct multilevel modelling analyses. However, results for group-level effects should be interpreted with consideration of the low ICC values.

We attempted to estimate coefficients using Full Information Maximum Likelihood (FIML), which retains all cases in the sample, but most models for Aim 2 would not converge owing to the complexity of the model and limitations in level 2 units and variance. Because using multilevel modelling was important given the nested structure of the data, and our primary hypotheses which involved examination of group-level social connections, coefficients were estimated using Restricted Maximum Likelihood (REML), which employs list-wise deletion. Thus, the sample size varies across analyses and is provided in footnotes for Tables 4 and 5. We did not complete an a priori power calculation to determine sample size, because this was a secondary data analysis of an existing data set.

#### Aim 1

We cross-sectionally assessed how social network centrality (in-degree, out-degree, in-eigenvector, and out-eigenvector) and reciprocated friendship ties were associated with participants’ high school GPA, psychological adjustment (depression/anxiety/hostility), and institutional attachment at the beginning of the school year (baseline). Importantly, models contained both the participant’s individual social network measure and the learning community group mean of the social network measure, to evaluate the incremental contribution of each after considering the variance explained by the other. Covariates at level 1 included participant age, gender, and family income. Covariates at level 2 included the group means of participant age and income, and the number of participants who completed the sociometric measure (see Appendix for the equations used).

#### Aim 2

We longitudinally assessed if greater social network centrality and more reciprocated friendship ties at the beginning of the academic year (baseline) were associated with higher first-year GPA, psychological adjustment, and institutional attachment outcomes at the end of the academic year (follow-up), after covarying the same adjustment measure at baseline. These models contained the same level 1 and level 2 demographic covariates as in Aim 1. In longitudinal analyses predicting university GPA, we additionally adjusted for the number of credits the participant took during the school year at both the individual and group level. Models contained both the participant’s individual social network measure and the learning community group mean of the social network measure, to evaluate the incremental contribution of each after considering the variance explained by the other (see Appendix for the equations used).

## Results

### Descriptive statistics

Table 2 displays the means and standard deviations of our study measures. Among the social network measures, there was a strong, positive correlation between in-degree and out-degree centrality and between out-degree and out-eigenvector centrality; and a moderate positive correlation between out-eigenvector centrality and out-degree centrality (Table 3).

**Table 3.** Bivariate Pearson’s correlations among social network centrality measures.

Variable	In-degree	Out-degree	In-eigenvector	Out-eigenvector
In-degree	-			
Out-degree	.67	-		
In-eigenvector	.56	.32	-	
Out-eigenvector	.37	.64	.55	-

*Note.* The size of the learning community was partialled out.

### Aim 1

Table 4 displays the cross-sectional analyses testing associations between social network measures and adjustment at baseline, when holding other predictors and random effects constant.

**Table 4.** Cross-sectional associations between social network characteristics at the individual and group level with individuals’ adjustment^a^.

Variable	Level	*B* (SE)	95% CI	*p*	ICC
High school GPA^b^					
In-degree	Within^c^	−0.22 (0.19)	−0.59, 0.15	.248	
	Between^d^	<0.01 (0.30)	−0.59, 0.59	.995	
					.01
Out-degree	Within	−0.05 (0.15)	−0.35, 0.25	.750	
	Between	<0.01 (0.30)	−0.59, 0.59	.999	
					.01
In-eigenvector	Within	−0.70 (2.94)	−6.48, 5.08	.812	
	Between	−1.03 (6.03)	−12.87, 10.82	.865	
					.01
Out-eigenvector	Within	−1.39 (2.61)	−6.53, 3.75	.596	
	Between	−1.25 (12.96)	−26.72, 24.22	.924	
					.01
Reciprocated friendship ties	Within	−0.18 (0.20)	−0.57, 0.21	.359	
	Between	−0.02 (0.33)	−0.67, 0.63	.951	
					.01
Depression					
In-degree	Within	<0.01 (0.02)	−0.03, 0.03	.971	
	Between	−0.01 (0.03)	−0.06, 0.04	.644	
					<.01
Out-degree	Within	−0.02 (0.01)	−0.05, <0.01	.066	
	Between	−0.01 (0.03)	−0.06, 0.04	.707	
					<.01
In-eigenvector	Within	0.16 (0.26)	−0.36, 0.68	.551	
	Between	0.03 (0.54)	−1.03, 1.08	.961	
					<.01
Out-eigenvector	Within	−0.23 (0.23)	0.68, 0.23	.330	
	Between	−0.51 (1.11)	−2.70, 1.67	.646	
					<.01
Reciprocated friendship ties	Within	−0.03 (0.02)	−0.07, <0.01	.066	
	Between	−0.02 (0.03)	−0.08, 0.04	.465	
					<.01
Anxiety					
In-degree	Within	<0.01 (0.02)	−0.03, 0.03	.769	
	Between	0.04 (0.02)	−0.01, 0.08	.137	
					<.01
Out-degree	Within	−0.02 (0.01)	−0.04, <.01	.105	
	Between	0.04 (0.02)	−0.01, 0.08	.121	
					<.01
In-eigenvector	Within	−0.11 (0.24)	−0.58, 0.37	.660	
	Between	0.18 (0.49)	−0.77, 1.14	.705	
					<.01
Out-eigenvector	Within	−0.31 (0.21)	−0.73, 0.10	.136	
	Between	0.14 (0.99)	−1.81, 2.09	.888	
					<.01
Reciprocated friendship ties	Within	−0.03 (0.02)	−0.06, <0.01	.055	
	Between	0.03 (0.03)	−0.02, 0.08	.196	
					<.01
Hostility					
In-degree	Within	−0.01 (0.01)	−0.04, 0.01	.269	
	Between	<0.01 (0.02)	−0.04, 0.04	.947	
					.04
Out-degree	Within	−0.02 (0.01)	−0.03, <0.01	.111	
	Between	<0.01 (0.02)	−0.04, 0.05	.911	
					.04
In-eigenvector	Within	−0.15 (0.19)	−0.53, 0.22	.426	
	Between	0.08 (0.42)	−0.75, 0.91	.857	
					.03
Out-eigenvector	Within	−0.24 (0.17)	−0.57, 0.09	.149	
	Between	0.24 (0.90)	−1.54, 2.02	.792	
					.04
Reciprocated friendship ties	Within	**−0.03 (0.01)**	**−0.06, −0.01**	.009**	
	Between	−0.02 (0.02)	−0.06, 0.03	.505	
					.04
Institutional attachment					
In-degree	Within	**0.05 (0.02)**	**<0.01, 0.10**	.032*	
	Between	**0.09 (0.04)**	**0.02, 0.16**	.017*	
					<.01
Out-degree	Within	**0.07 (0.02)**	**0.03, 0.10**	<.001***	
	Between	**0.08 (0.04)**	**0.01, 0.16**	.022*	
					<.01
In-eigenvector	Within	0.55 (0.37)	−0.18, 1.27	.139	
	Between	1.38 (0.75)	−0.09, 2.85	.066	
					<.01
Out-eigenvector	Within	**0.84 (0.32)**	**0.21, 1.48**	.009**	
	Between	0.91 (1.56)	−2.15, 3.98	.561	
					.01
Reciprocated friendship ties	Within	**0.09 (0.02)**	**0.04, 0.13**	<.001***	
	Between	**0.10 (0.04)**	**0.02, 0.18**	.013*	
					<.01

*Note.* Only the associations between out-degree centrality and, reciprocated friendship ties, and institutional attachment at the within-groups level remained after Bonferroni correction.

*Note.* The use of bolding indicates results where p<.05.

*Note.* Several of our models had a singularity error and thus the random effects are unreliable. However, all models still converged.

*Note.* Covariates in all models included age, gender, income, and learning community size.

*p < .05 before Bonferonni correction; **p < .01 before Bonferonni correction; ***p < .005 after Bonferonni correction (the *p*-value cutoff for Bonferonni correction was 0.05/25 = 0.002 for each outcome).

^a^The sample size across analyses ranged from *n* = 424 to *n* = 446.

^b^High-school GPA was self-reported at baseline but would have been determined prior to the study (and thus before the other network items).

^c^Within Groups refers to the level of the individual (e.g., individuals associations within a learning community).

^d^Between Groups refers to the group mean level (e.g., differences between learning communities).

#### In-degree/out-degree centrality

There were two associations at the individual level between in-degree/out-degree centrality and adjustment. Specifically, individuals with higher in-degree centrality than their learning community average had higher institutional attachment (*B* = 0.05, 95% CI [<0.01, 0.10]). As well, individuals who had higher out-degree centrality than their learning community average had higher institutional attachment (*B* = 0.07, 95% CI [0.03, 0.10]). This suggests that individuals who sent or received more friendship nominations had a higher sense of belonging at their institution concurrently.

There were two associations at the learning community level. The learning community mean-level in-degree centrality (*B* = 0.09, 95% CI [0.02, 0.16]) and out-degree centrality (*B* = 0.08, 95% CI [0.01, 0.16]) were associated with participants’ higher institutional attachment. This suggests that in learning communities where members had more incoming or outgoing friendship ties on average, participants had a greater sense of belonging at their institution than in learning communities with fewer incoming or outgoing friendship ties, after accounting for participants’ own individual numbers of ties.

#### In-eigenvector/out-eigenvector centrality

There was one association at the individual level between in-eigenvector/out-eigenvector centrality and adjustment. Individuals with higher out-eigenvector centrality than their learning community average (who sent more friendship ties to highly connected members of their learning community) had higher institutional attachment concurrently (*B* = 0.84, 95% CI [0.21, 1.48]). There were no associations at the learning community level for in-eigenvector or out-eigenvector centrality.

#### Reciprocated friendship

There were two associations at the individual level between reciprocated friendship ties and adjustment. Individuals with more reciprocated friendship ties than their learning community average of reciprocated friendships had lower hostility (*B* = −0.03, 95% CI [−0.06, −0.01]) and higher institutional attachment concurrently (*B* = 0.09, 95% CI [0.04, 0.13]).

At the learning community level, there was one association. The learning community mean-level of reciprocated friendship ties was associated with participants’ higher institutional attachment (*B* = 0.10, 95% CI [0.02, 0.18]). This suggests that participants in learning communities where members had more mutually endorsed friendships, after accounting for participants’ own levels of mutually endorsed friendships, had a greater sense of attachment to their university.

### Aim 2

Table 5 depicts the longitudinal analysis testing baseline social network variables as predictors of follow-up adjustment, after accounting for original levels of baseline adjustment and holding other predictors and random effects constant.

**Table 5.** Longitudinal associations between individual and group level social network measures at baseline and individuals’ adjustment at follow-up^a^.

Variable	Level	*B* (SE)	95% CI	*p*	ICC
First-year GPA^b^					
In-degree	Within^c^	−0.25 (0.26)	−0.76, 0.26	.338	
	Between^d^	<0.01 (0.52)	−1.02, 1.02	.999	
					.09
Out-degree	Within	−0.24 (0.21)	−0.65, 0.17	.246	
	Between	−0.02 (0.52)	−1.04, 1.01	.977	
					.09
In-eigenvector^d^	Within	−3.60 (3.98)	−11.43, 4.22	.366	
	Between	**23.85 (8.90)**	**6.35, 41.35**	.009**	
					.07
Out-eigenvector	Within	−3.26 (3.57)	−10.28, 3.75	.361	
	Between	18.01 (21.51)	−60.33, 24.30	.406	
					.09
Reciprocated friendship ties	Within	−0.24 (0.27)	−0.77, 0.30	.387	
	Between	0.23 (0.56)	−0.88, 1.34	.683	
					.09
Depression					
In-degree	Within	0.02 (0.02)	−0.02, 0.05	.400	
	Between	<0.01 (0.03)	−0.07, 0.06	.880	
					.02
Out-degree	Within	0.01 (0.01)	−0.02, 0.04	.570	
	Between	−0.01 (0.03)	−0.07, 0.06	.876	
					.02
In-eigenvector	Within	0.09 (0.28)	−0.47, 0.65	.756	
	Between	−0.37 (0.68)	−1.71, 0.96	.584	
					.02
Out-eigenvector	Within	0.22 (0.25)	−0.28, 0.72	.381	
	Between	1.57 (1.26)	−0.91, 4.04	.222	
					.02
Reciprocated friendship ties	Within	0.02 (0.02)	−0.01, 0.06	.195	
	Between	<0.01 (0.04)	−0.07, 0.07	.909	
					.02
Anxiety					
In-degree	Within	0.01 (0.02)	−0.02, 0.05	.512	
	Between	0.01 (0.03)	−0.04, 0.07	.664	
					<.01
Out-degree	Within	<0.01 (0.01)	−0.02, 0.03	.739	
	Between	0.01 (0.03)	−0.04, 0.07	.665	
					<.01
In-eigenvector	Within	0.37 (0.27)	−0.15, 0.90	.162	
	Between	−0.02 (0.60)	−1.41, 0.95	.702	
					<.01
Out-eigenvector	Within	**0.52 (0.24)**	**0.05, 0.99**	.029*	
	Between	0.89 (1.09)	−1.25, 3.04	.414	
					<.01
Reciprocated friendship ties	Within	0.01 (0.02)	−0.02, 0.05	.425	
	Between	<0.01 (0.03)	−0.06, 0.06	.967	
					<.01
Hostility					
In-degree	Within	0.01 (0.02)	−0.02, 0.04	.381	
	Between	−0.03 (0.02)	−0.08, 0.02	.260	
					<.01
Out-degree	Within	<0.01 (0.01)	−0.02, 0.03	.786	
	Between	−0.03 (0.02)	−0.08, 0.02	.262	
					<.01
In-eigenvector	Within	−0.10 (0.23)	−0.55, 0.36	.678	
	Between	**−1.03 (0.52)**	**−2.07, −<0.01**	.049*	
					<.01
Out-eigenvector	Within	0.10 (0.21)	−0.32, 0.51	.650	
	Between	0.46 (0.97)	−1.45, 2.36	.637	
					<.01
Reciprocated friendship ties	Within	0.01 (0.02)	−0.02, 0.04	.596	
	Between	−0.03 (0.03)	−0.08, 0.02	.246	
					<.01
Institutional attachment					
In-degree	Within	−0.01 (0.02)	−0.05, 0.04	.744	
	Between	0.02 (0.04)	−0.06, 0.11	.585	
					.02
Out-degree	Within	0.02 (0.02)	−0.02, 0.05	.389	
	Between	0.02 (0.04)	−0.06, 0.11	.615	
					.02
In-eigenvector	Within	−0.36 (0.35)	−1.05, 0.34	.313	
	Between	**1.87 (0.83)**	**0.24, 3.50**	.026*	
					.01
Out-eigenvector	Within	−0.25 (0.32)	−0.88, 0.37	.429	
	Between	1.90 (1.57)	−1.22, 5.02	.240	
					.02
Reciprocated friendship ties	Within	0.01 (0.02)	−0.04, 0.05	.834	
	Between	0.01 (0.05)	−0.08, 0.10	.782	
					.02

*Note.* None of the associations remained after Bonferonni correction.

*Note.* The use of bolding indicates results where p<.05.

*Note.* Several of our models had a singularity error and thus the random effects are unreliable. However, all models still converged.

*Note.* Covariates in all models included age, gender, income, learning community size, and the adjustment variable at baseline. Where first-year GPA is an outcome, the number of course credits attempted was also included.

*p < .05 before Bonferonni correction; **p < .01 before Bonferonni correction; ***p < .005 after Bonferonni correction (the *p*-value cutoff for Bonferonni correction was 0.05/25 = 0.002 for each outcome).

^a^The sample size across analyses ranged from *n* = 350 to *n* = 358.

^b^Within Groups refers to the level of the individual (e.g., individuals associations within a learning community).

^c^Between Groups refers to the group mean level (e.g., differences between learning communities).

^d^The association between in-eigenvector centrality and first-year GPA should be interpreted with caution due to small group sizes. As in-eigenvector centrality is a weighted score based on how well-connected one’s connections are, it is particularly sensitive to disparities in group sizes.

#### In-degree/out-degree centrality

These network centrality measures were not significant predictors of any outcome variable at the individual level or the learning community level.

#### In-eigenvector/out-eigenvector centrality

At the individual level, there was one relationship between in-eigenvector/out-eigenvector centrality and adjustment. Individuals with higher out-eigenvector centrality than their learning community average at baseline had higher anxiety at follow-up (*B* = 0.52, 95% CI [0.05, 0.99]).

At the learning community level, network centrality predicted three outcomes. The learning community mean in-eigenvector centrality was associated with participants’ higher first-year GPA (*B* = 23.85, 95% CI [6.35, 41.35]) and institutional attachment (*B* = 1.87, 95% CI [0.24, 3.50]), and lower hostility (*B* = −1.03, 95% CI [−2.07, −<0.01]). This suggests that being in a learning community that had a higher level of incoming friendship ties among well-connected individuals at baseline was associated with participants being more academically successful, less hostile, and reporting greater institutional attachment, at follow-up. Importantly, these associations existed after accounting for participants’ adjustment levels at baseline, and participants’ individual incoming friendship ties among well-connected individuals.

#### Reciprocated friendship

At the individual level, as well as at the learning community level, reciprocated friendship ties at baseline were not associated with adjustment at follow-up.

## Discussion

In a sample of 717 first-year international undergraduate students nested in 59 learning communities, we evaluated associations between individual-level social connections (the one-on-one ties individuals have with others in their group) and the group-level social milieu (the overall pattern of social connections within the group), with indicators of individuals’ adjustment. We found that individuals with better social network integration tended to have a greater sense of attachment to their university and lower hostility, and that being part of a well-connected group was associated with better adjustment across an academic year, even when adjusting for individuals’ own level of connectedness. This work adds to an existing body of literature that advances the importance of social connections during a major life transition and emphasizes the benefits of fostering a positive, socially-connected group or community environment.

### Cross-sectional analyses

First, in cross-sectional analyses, we evaluated if social network measures were associated with psychological adjustment at baseline. At the individual level, there was evidence that three measures of network centrality (in-degree, out-degree, and out-eigenvector), as well as reciprocated friendship ties, were associated with higher institutional attachment. Having more reciprocated friendship ties was also associated with lower hostility. More incoming, outgoing, or reciprocated ties may foster a sense of belonging (Levasseur et al., 2017) which is a major component of institutional attachment. Reciprocated friendship ties may indicate stronger relationships, and such close ties could provide more resources and support (Vaquera & Kao, 2008) which may in turn protect against hostility. Conversely, it could be that individuals who are more hostile have more difficulty forming closer friendships.

At the group level, we observed a cross-sectional association between the learning community having more overall incoming friendship nominations, outgoing friendship nominations, and reciprocated friendship nominations (above and beyond individuals’ own connections) and higher institutional attachment of the individuals in that group. Networks with more social integration may provide members with a sense of collective pride, which fosters a positive and welcoming social environment. This kind of social environment may in turn facilitate a greater sense of connection and belonging to one’s institution where these friendships are embedded. Dai and Shi (2022) found that individuals who simply observed supportive interactions in online communities were influenced vicariously by these interactions, especially when they identified with those involved in the interaction. This suggests that individuals in well-connected groups may benefit from observing positive exchanges within their learning communities, even if they were not directly involved in those exchanges.

### Longitudinal analyses

Second, we longitudinally assessed if better social network centrality or reciprocated friendship ties at the beginning of the academic year (baseline) were associated with better adjustment outcomes at the end of the academic year (follow-up), after accounting for baseline adjustment. Contrary to our expectations, at the individual level, participants with higher out-eigenvector centrality had higher anxiety at follow-up. Post-hoc, we speculate that nominating many well-connected peers as friends may reflect that individual desiring relationships with high-status peers, as out-eigenvector centrality was calculated independently of whether those peers reciprocate the ties. Indeed, recent work in an adolescent sample found that sending out more unreciprocated friendship nominations was associated with a stronger desire for more friendships and also greater symptoms of anxiety (Walsh et al., 2023), which the authors attribute to having maladaptive goals that emphasize social status.

At the group level, we found that higher learning community levels of in-eigenvector centrality were associated with individuals’ higher first-year GPA, lower hostility, and higher institutional attachment at the end of the academic year. In other words, students tended to be more academically successful, less hostile, and expressed a greater sense of belonging and fit at university at the end of their first year, when they were part of learning communities in which group members were connected to other well-connected individuals in the network. These associations were found after accounting for individuals’ own levels of connectedness as well as their adjustment at the beginning of the year. Interestingly, while social network centrality was associated with a greater sense of belonging in cross-sectional analyses for all but in-degree and group level out-eigenvector centrality, these associations between social network centrality and sense of belonging did not hold at follow-up (with the exception of group-level in-eigenvector centrality). One possible explanation is that having many friends at the beginning of the school year is important for students’ sense of belonging, but that over the long term, belongingness may depend more on whether the friendships are meeting students’ needs (e.g., providing emotional support) relative to how many friendships they have. Alternatively, it could be that friendships in the Jump Start program were not maintained.

Being a part of a tight-knit, interconnected network likely offers a strong sense of community, which may translate into a feeling of “we’re in this together” that fosters a larger sense of belonging and attachment to the institution (Roffey, 2013). Well-connected individuals in the network might be particularly effective at gathering resources and information (e.g., academic support; information about upcoming social events) from diverse sources and facilitating the flow of information throughout the network. These well-connected individuals may be especially impactful when the group is cohesive. Enhanced knowledge and access to support and resources may help individual members of the network thrive both academically and socially in their first year at university.

### Importance of the group social environment

Psychology’s tendency to emphasize individual-level social connections may stem from its adoption of a medical model to explain adjustment. Mental illnesses are diagnosed within individuals. Reasons for poor (or good) adjustment are located within the individual; treatment is largely targeted toward individuals rather than social systems and structures. The current study adds to the growing literature about how the larger social context in which individuals operate is fundamentally linked to their adjustment. Crucially, we obtained these findings in models that accounted for individual-level social connections, emphasizing the importance of building a sense of community within groups. Our findings align with social identity theory, which underscores that social groups can have profound influences on individuals’ behavior and attitudes (Tajfel & Turner, 1986). When group members are more connected (i.e., have more friendship ties and more interactions), this may encourage individuals to identify more strongly with the group (Graupensperger et al., 2020), which in turn may magnify group-level influences on individuals’ adjustment. Therefore, when considering social connection as a health asset, it is important to look beyond the individual and consider the groups in which they are situated.

Some ways to increase cohesion within groups include increasing intergroup contact, fostering a positive group identity, having individuals participate in group goal-setting activities, and developing group members’ self-efficacy (see Orazani et al., 2023 for a review). Because group leaders can be particularly effective at establishing group norms, interventions that influence leaders’ behaviors may be especially impactful. For instance, when group leaders cooperated in a public good dilemma game, group members tended to cooperate as well even in the absence of the group leader (Harrell, 2019). Simply increasing opportunities for group members to socialize and observe each others’ interactions may also foster cohesion; indeed, merely observing members of one’s group having positive interactions can promote a sense of connectedness (White et al., 2021). Even in situations where direct face-to-face social contact may not be feasible, online interventions can promote group adjustment (see Forrest & Bruner, 2017; Haslam, 2018 for examples of successful online interventions in youth and young adults). Thus, a multitude of avenues exist to enhance the sense of community within groups. University administrators aiming to increase cohesion in their schools could consider creating an orientation program such as Jump Start, or identifying existing social communities such as dormitory floors, campus clubs, or study groups within which building connection could be made an explicit goal. For example, the administration could provide support for activities where students work together to succeed and model inclusivity and kindness.

### Limitations and future directions

These findings should be considered in the context of the strengths and limitations of our study. Importantly, we acknowledge that pathways between social network centrality with adjustment are likely to be bidirectional. This study found cross-sectional associations between social network measures and adjustment at baseline, and some potential for baseline social network measures to predict adjustment at follow-up. However, we did not test the potential for adjustment at baseline to predict social network measures, either cross-sectionally or longitudinally, and we relied on self-reported measures which could be subject to bias. Future work should use techniques such as random intercept cross-lagged panel modeling, with network measures and adjustment measures collected at multiple timepoints across a period of time, to elucidate potential bidirectional pathways. Further, in all observational correlational and longitudinal designs, it is possible that factors beyond the predictors and control variables measured at baseline contribute to the outcomes at follow-up. Future work using experimental designs and interventions may help establish the robustness of these associations.

Our group-level effects must also be interpreted in light of the low ICC values for many models. Given that there was not much group-level variance to explain for some outcome variables, any statistically significant group-level effects that we found are likely to have relatively small effect sizes. Although this potentially limits the practical magnitude of the group-level findings, it may still be useful for university administrators to support socially connected environments for entering students. A policy change by university administrators, even if it has a small effect size in shifting the adjustment of students on average, can have practical importance if it reaches all students. Indeed, many universal interventions operate on the same principle.

Participants in our study could only nominate other individuals from their learning communities as friends, but it is likely that students developed friendships with individuals not in our study who may have played a role in their adjustment. Using a sociometric survey has the strength of not relying solely on individuals’ reports of their own friendships, but the sociometric method requires a defined group in which to administer it. Given that friendships can be unilateral (e.g., one’s in-degree centrality is not equal to their out-degree centrality), allowing both members of a dyad to report on their connections to each other provides a more complete picture of their relationship.

We used a sample of incoming students, most of whom had moved away from home to attend university, but the extent to which this generalizes to other individuals undergoing life transitions remains to be seen. These findings may be unique to emerging adults transitioning to university, as group norms may be particularly malleable during this period. Similarly, because our study was conducted in a single university, results may not generalize to other contexts. Future work should explore group-level effects of social connection during other life transitions, and within different group and cultural contexts (e.g., workplaces, neighborhoods).

While our study included international students from around the world, we did not explore how students’ identities may play a role in adjustment. Our demographic data contained only binary categories for gender and did not capture other identities such as sexual orientation, race/ethnicity, or whether one has a disability. Future work should consider intersectional approaches to better understand how identity may relate to social connection and adjustment.

Finally, an important future direction for understanding how social connections relate to adjustment is exploring individual differences that may promote social integration. Personal qualities such as self-esteem, social skills, and personality differences may contribute to successful integration into new social groups during a life transition. Similarly, students’ working models of relationships (e.g., the expectation that other people will be there for them) and feelings of self-efficacy may influence how successful they are in developing supportive social relationships during the transition to university, which may in turn, contribute to their adjustment over time. Future work should explore social relationships as a mediator for adjustment in individuals undergoing life transitions.

Given that the learning community average level of social network measures mattered for students’ individual-level adjustment, an important next step is research into factors that contribute to the social connectivity of groups. In addition to the research on interventions discussed above, one possibility is that the connectedness of groups is influenced by certain highly-influential individuals in the network who facilitate the spread of norms throughout their groups (Paluck & Shepherd, 2012). Perhaps such individuals are even more important in newly formed groups. Or, perhaps there are contextual factors about the Jump Start learning communities that are especially effective in catalyzing new connections or strengthening existing ones. Each learning community had two orientation leaders and a faculty fellow who interacted with the group for several hours each day. These individuals may have a particularly strong influence on the overall group social milieu, similar to the influence of elementary and secondary school teachers on their classroom climate (Hamre & Pianta, 2005; Mikami et al., 2012). Further research on the factors that promote group-level social connection may eventually serve as a solid foundation for creating interventions.

## Conclusion

The present research suggests that in addition to an individual’s one-on-one social connections with others, being in a group with many overall social connections may incrementally facilitate adjustment. This work offers support for programs and activities that bolster positive social environments. Given that mental health challenges such as depression and anxiety are increasingly prevalent in university students (Heumann et al., 2024), university administrators and others who are invested in student well-being may consider ways to increase both students’ individual-level social connections with their peers, as well as a positive and connected group climate overall. However, more work is needed to understand predictors of group-level connection and the mechanisms through which it benefits well-being.

## Supplemental Material

**Supplemental Material -** Better together: Group-level social dynamics predict individuals’ psychological adjustment during a major life transition Supplemental Material for Better together: Group-level social dynamics predict individuals’ psychological adjustment during a major life transition by Marisa A. Nelson, Julia S. Nakamura, Frances S. Chen, Amori Yee Mikami in Journal of Social and Personal Relationships.

## Data Availability

The data used in this study are not openly available. This study is a secondary data analysis of an existing dataset. During collection, we did not get participants’ permission to post it publicly. We can share the anonymized data with researchers who have received ethics board approval to receive it upon request.*
