# Supplementary material for: Better together: Group-level social dynamics predict individuals’ psychological adjustment during a major life transition
Source: J Soc Pers Relat. 2025 Nov 24;43(5):1313–38. doi: 10.1177/02654075251401692 (PMC13044484; doi:10.1177/02654075251401692)
Supplement: Supplemental Material - Better together: Group-level social dynamics predict individuals’ psychological adjustment during a major life transition [file sj-pdf-1-spr-10.1177_02654075251401692.pdf]

**Appendix 1**

*Equations for Research Questions 1 and 2*

Research question 1:

Level 1: $Y_{ij} = \beta_{0j} + \beta_{1j}$ (baseline social network measure) + $\beta_{2j}$ (age) + $\beta_{3j}$ (gender) + $\beta_{4j}$ (income) + $r_{ij}$

Level 2: $\beta_{0j} = \gamma_{00} + \gamma_{01}$ (group mean of baseline social network measure) + $\gamma_{02}$ (learning community size) + $\gamma_{03}$ (group mean of covariates) + $u_{0j}$

$\beta_{1j} = \gamma_{10}$

$\beta_{2j} = \gamma_{20}$

At level 1, the participant adjustment measure at baseline ($Y_{ij}$) was the criterion variable. This was predicted by the intercept ($\beta_{0j}$), the participant's baseline social network measure ($\beta_{1j}$), the covariates of participant age ($\beta_{2j}$), gender ($\beta_{3j}$), and income ($\beta_{4j}$), and the residual variance ($r_{ij}$). At level 2, we entered the learning community group mean of the social network measure ($\gamma_{01}$), the number of participants in the learning community who completed the sociometric measure ($\gamma_{02}$), and the learning community group means of participant age ($\gamma_{03}$) and income ($\gamma_{04}$), and the residual variance ($u_{0j}$), as predictors of the level 1 intercept.

Research question 2:

Level 1: $Y_{ij} = \beta_{0j} + \beta_{1j}$ (baseline social network measure) + $\beta_{2j}$ (baseline adjustment) +

$\beta_{3j}$ (age) + $\beta_{4j}$ (gender) + $\beta_{5j}$ (income) + $r_{ij}$

Level 2: $\beta_{0j} = \gamma_{00} + \gamma_{01}$ (group mean of baseline social network measure) + $\gamma_{02}$ (group

mean of baseline adjustment) + $\gamma_{03}$ (learning community size) + $\gamma_{04}$ (group mean of age) + $\gamma_{05}$ (group mean of income) + $u_{0j}$

$\beta_{1j} = \gamma_{10}$

$\beta_{2j} = \gamma_{20}$

$\beta_{3j} = \gamma_{30}$

At level 1, participant adjustment at follow-up ($Y_{ij}$) was the criterion variable. This was predicted by the intercept ($\beta_{0j}$), the participant's baseline social network measure ($\beta_{1j}$), the covariates of the baseline measure of participant adjustment ($\beta_{2j}$), participant age ($\beta_{3j}$), gender ($\beta_{4j}$), and income ($\beta_{5j}$), and the residual variance ($r_{ij}$). At level 2, we entered the learning community group mean of the baseline social network measure ($\gamma_{01}$), the group mean of the baseline adjustment measure ($\gamma_{02}$), the number of participants in the learning community who completed the sociometric measure ($\gamma_{03}$), and the learning community group means of participant age ($\gamma_{04}$) and the group mean of income ($\gamma_{05}$), and the residual variance ($u_{0j}$), as predictors of the level 1 intercept. For the outcome measure of first year GPA, we included the number of credits attempted as covariates at both level 1 (number of credits the participant attempted) and level 2 (learning community mean of credits attempted).

**Appendix Table 1**

*Cross-Sectional Associations Between In-Degree Centrality at the Individual and Group Level with Individuals' Adjustment (Full Covariates)*[a]

| Effect | High School GPA[b] | | | Depression | | | Anxiety | | | Hostility | | | Institutional attachment | | |
|---|---|---|---|---|---|---|---|---|---|---|---|---|---|---|---|
| | *b* (SE) | *95% CI* | *p* | *b* (SE) | *95% CI* | *p* | *b* (SE) | *95% CI* | *p* | *b* (SE) | *95% CI* | *p* | *b* (SE) | *95% CI* | *p* |
| Intercept | **130.14** | **76.83,** | **<.001*** | 1.67 (2.34) | -2.93, 6.27 | .480 | 0.88 (2.11) | -3.28, 5.03 | .678 | 0.76 (1.96) | -3.09, 4.61 | .701 | **7.35 (3.24)** | **0.98, 13.71** | **.024*** |

| | | | | | | | | | | | | | | | |
|---|---|---|---|---|---|---|---|---|---|---|---|---|---|---|---|
| | **(27.12)** | **183.45** | ** | | | | | | | | | | | | |
| In-degree Centrality (w)[c] | -0.22 (0.19) | -0.59, 0.15 | .248 | <0.01 (0.02) | -0.03, 0.03 | .971 | <0.01 (0.02) | -0.03, 0.03 | .769 | -0.01 (0.01) | -0.04, 0.01 | .269 | **0.05 (0.02)** | **0.00, 0.10** | **.032*** |
| In-degree Centrality (b)[d] | <0.01 (0.30) | -0.59, 0.59 | .995 | -0.01 (0.03) | -0.06, 0.04 | .644 | 0.04 (0.02) | -0.01, 0.08 | .137 | <0.01 (0.02) | -0.04, 0.04 | .947 | **0.09 (0.04)** | **0.02, 0.16** | **.017*** |
| Age (w)[c] | -0.19 (0.56) | -1.29, 0.92 | .741 | 0.06 (0.04) | -0.01, 0.14 | .108 | 0.05 (0.04) | -0.02, 0.12 | .140 | -0.03 (0.03) | -0.09, 0.03 | .300 | -0.07 (0.06) | -0.18, 0.04 | .222 |
| Age (b)[d] | -2.57 (1.45) | -5.42, 0.28 | .086 | <0.01 (0.12) | -0.24, 0.25 | .977 | 0.04 (0.11) | -0.18, 0.26 | .710 | 0.05 (0.10) | -0.16, 0.25 | .661 | -0.02 (0.17) | -0.36, 0.32 | .895 |
| Gender | 0.48 (0.87) | -1.22, 2.19 | .578 | 0.14 (0.08) | -0.02, 0.29 | .082 | **0.23 (0.07)** | **0.09, 0.37** | **.001 **** | -0.02 (0.06) | -0.13, 0.09 | .753 | 0.01 (0.11) | -0.22, 0.21 | .957 |
| Learning Community Size | 0.12 (0.14) | -0.16, 0.39 | .407 | 0.01 (0.01) | -0.01, 0.04 | .286 | -0.01 (0.01) | -0.03, 0.01 | .264 | -0.01 (0.01) | -0.03, 0.01 | .332 | -0.02 (0.02) | -0.05, 0.02 | .391 |
| Income (w)[c] | 0.22 (0.46) | -0.69, 1.13 | .632 | -0.05 (0.04) | -0.13, 0.04 | .279 | -0.04 (0.04) | -0.12, 0.03 | .286 | -0.05 (0.03) | -0.11, 0.01 | .080 | 0.04 (0.06) | -0.07, 0.16 | .471 |
| Income (b)[d] | -1.47 (0.97) | -3.38, 0.43 | .131 | 0.06 (0.09) | -0.11, 0.24 | .465 | 0.04 (0.08) | -0.12, 0.19 | .650 | 0.07 (0.07) | -0.07, 0.20 | .320 | -0.04 (0.12) | -0.27, 0.20 | .768 |
| ICC[e] | | .01 | | | <.01 | | | <.01 | | | .04 | | | <.01 | |

*Note*. Some of our model estimations resulted in a singularity error, and thus the random effects are unreliable. However, all models still converged. Intra-class correlations (ICCs) were low for these models (<5%), and for several of our dependent variables, the random effects intercept variance was 0. However, because group-level effects are of central theoretical interest to us, we decided to retain and report two levels in these models.

[a]The sample size across analyses ranged from *n* = 424 to *n* = 446.

[b]High school GPA was self-reported at baseline but would have been determined prior to the study (and thus before the other network items).

[c](w) represents within-group effects, which refers to the level of the individual (e.g., individuals associations within a learning community).

[d](b) represents between-group effects, which refers to the group mean level (e.g., differences between learning communities).

[e]Unadjusted intra-class correlations

*p < 0.05; **p < 0.01; ***p<0.001

## Appendix Table 2

*Cross-Sectional Associations Between Out-Degree Centrality at the Individual and Group Level with Individuals' Adjustment (Full Covariates)*[a]

| Effect | High School GPA[b] | | | Depression | | | Anxiety | | | Hostility | | | Institutional attachment | | |
|---|---|---|---|---|---|---|---|---|---|---|---|---|---|---|---|
| | *b* (SE) | *95% CI* | *p* | *b* (SE) | *95% CI* | *p* | *b* (SE) | *95% CI* | *p* | *b* (SE) | *95% CI* | *p* | *b* (SE) | *95% CI* | *p* |
| Intercept | **129.21 (27.15)** | **75.83, 182.59** | **<.001 *** ** | 1.54 (2.37) | -3.11, 6.20 | .519 | 0.76 (2.11) | -3.39, 4.90 | .720 | 0.63 (1.97) | -3.24, 4.50 | .751 | **7.86 (3.21)** | **1.55, 14.17** | **.015 *** |
| Out-degree Centrality (w)[c] | -0.05 (0.15) | -0.35, 0.25 | .750 | -0.02 (0.01) | -0.05, 0.00 | .066 | -0.02 (0.01) | -0.04, 0.00 | .105 | -0.02 (0.01) | -0.03, 0.00 | 0.111 | **0.07 (0.02)** | **0.03, 0.10** | **<.001 *** ** |
| Out-degree Centrality (b)[d] | <0.01 (0.30) | -0.59, 0.59 | .999 | -0.01 (0.03) | -0.06, 0.04 | .707 | 0.04 (0.02) | -0.01, 0.08 | .121 | <0.01 (0.02) | -0.04, 0.05 | .911 | **0.08 (0.04)** | **0.01, 0.16** | **.022 *** |
| Age (w)[c] | -0.18 (0.57) | -1.29, 0.93 | .753 | 0.06 (0.04) | -0.02, 0.14 | .148 | 0.05 (0.04) | -0.02, 0.12 | .179 | -0.03 (0.03) | -0.09, 0.02 | .253 | -0.05 (0.06) | -0.16, 0.06 | .337 |
| Age (b)[d] | -2.52 (1.45) | -5.37, 0.33 | .092 | 0.01 (0.13) | -0.24, 0.26 | .939 | 0.05 (0.11) | -0.17, 0.27 | .671 | 0.05 (0.11) | -0.15, 0.26 | .620 | -0.05 (0.17) | -0.38, 0.29 | .780 |
| Gender | 0.51 (0.87) | -1.20, 2.22 | .559 | 0.15 (0.08) | -0.01, 0.30 | .060 | **0.24 (0.07)** | **0.10, 0.38** | **.001*** ** | -0.01 (0.06) | -0.12, 0.10 | .863 | -0.04 (0.11) | -0.25, 0.17 | .694 |
| Learning Community Size | 0.12 (0.14) | -0.15, 0.40 | .394 | 0.01 (0.01) | -0.01, 0.04 | .297 | -0.01 (0.01) | -0.03, 0.01 | .259 | -0.01 (0.01) | -0.03, 0.01 | .337 | -0.01 (0.02) | -0.05, 0.02 | .375 |
| Income (w)[c] | 0.24 (0.46) | -0.67, 1.16 | .598 | -0.05 (0.04) | -0.13, 0.04 | .273 | -0.04 (0.04) | -0.12, 0.03 | .287 | -0.05 (0.03) | -0.11, 0.01 | .086 | 0.04 (0.06) | -0.08, 0.15 | .514 |
| Income (b)[d] | -1.47 (0.97) | -3.38, 0.43 | .132 | 0.06 (0.09) | -0.11, 0.24 | .464 | 0.04 (0.08) | -0.12, 0.19 | .645 | 0.07 (0.07) | -0.07, 0.20 | .319 | -0.04 (0.12) | -0.27, 0.20 | .759 |
| ICC[e] | | .01 | | | <.01 | | | <.01 | | | .04 | | | <.01 | |

*Note.* Some of our model estimations resulted in a singularity error, and thus the random effects are unreliable. However, all models still converged. Intra-class correlations (ICCs) were low for these models (<5%), and for several of our dependent variables, the random effects intercept variance was 0. However, because group-level effects are of central theoretical interest to us, we decided to retain and report two levels in these models.

[a]The sample size across analyses ranged from *n* = 424 to *n* = 446.

[b]High school GPA was self-reported at baseline but would have been determined prior to the study (and thus before the other network items).

[c](w) represents within-group effects, which refers to the level of the individual (e.g., individuals associations within a learning community).

[d](b) represents between-group effects, which refers to the group mean level (e.g., differences between learning communities).

[e]Unadjusted intra-class correlations

*p < 0.05; **p < 0.01; ***p<0.001

## Appendix Table 3

*Cross-Sectional Associations Between In-Eigenvector Centrality at the Individual and Group Level with Individuals' Adjustment (Full Covariates)*[a]

| Effect | High School GPA[b] | | | Depression | | | Anxiety | | | Hostility | | | Institutional attachment | | |
|---|---|---|---|---|---|---|---|---|---|---|---|---|---|---|---|
| | *b* (SE) | *95% CI* | *p* | *b* (SE) | *95% CI* | *p* | *b* (SE) | *95% CI* | *p* | *b* (SE) | *95% CI* | *p* | *b* (SE) | *95% CI* | *p* |
| Intercept | **129.69 (26.66)** | **77.29, 182.09** | **<.001***** | 1.45 (2.29) | -3.06, 5.96 | .527 | 1.41 (2.08) | -2.69, 5.50 | .500 | 0.75 (1.92) | -3.02, 4.53 | .698 | **8.52 (3.21)** | **2.21, 14.83** | **.008 **** |
| In-Eigenvector Centrality (w)[c] | -0.70 (2.94) | -6.48, 5.08 | .812 | 0.16 (0.26) | -0.36, 0.68 | .551 | -0.11 (0.24) | -0.58, 0.37 | .660 | -0.15 (0.19) | -0.53, 0.22 | .426 | 0.55 (0.37) | -0.18, 1.27 | .139 |
| In-Eigenvector Centrality (b)[d] | -1.03 (6.03) | -12.87, 10.82 | .865 | 0.03 (0.54) | -1.03, 1.08 | .961 | 0.18 (0.49) | -0.77, 1.14 | .705 | 0.08 (0.42) | -0.75, 0.91 | .857 | 1.38 (0.75) | -0.09, 2.85 | .066 |
| Age (w)[c] | -0.17 (0.56) | -1.28, 0.94 | .769 | 0.06 (0.04) | -0.01, 0.14 | .104 | 0.05 (0.04) | -0.02, 0.12 | .148 | -0.03 (0.03) | -0.09, 0.03 | .303 | -0.07 (0.06) | -0.18, 0.04 | .189 |
| Age (b)[d] | -2.53 (1.42) | -5.33, 0.27 | .086 | 0.01 (0.12) | -0.23, 0.26 | .905 | 0.01 (0.11) | -0.21, 0.23 | .921 | 0.05 (0.10) | -0.16, 0.25 | .662 | -0.11 (0.17) | -0.45, 0.23 | .526 |
| Gender | 0.49 (0.87) | -1.22, 2.20 | .572 | 0.13 (0.08) | -0.02, 0.29 | .086 | **0.24 (0.07)** | **0.10, 0.37** | **.001***** | -0.02 (0.06) | -0.13, 0.09 | .755 | -0.01 (0.11) | -0.22, 0.21 | .963 |
| Learning Community Size | 0.11 (0.11) | -0.11, 0.34 | .333 | 0.01 (0.01) | -0.01, 0.03 | .336 | <.01 (0.01) | -0.02, 0.02 | .985 | -0.01 (0.01) | -0.03, 0.01 | .293 | 0.02 (0.01) | -0.01, 0.05 | .114 |
| Income (w)[c] | 0.24 (0.47) | -0.68, 1.15 | .610 | -0.04 (0.04) | -0.13, 0.04 | .297 | -0.04 (0.04) | -0.12, 0.03 | .280 | -0.05 (0.03) | -0.11, 0.01 | .080 | 0.04 (0.06) | -0.07, 0.16 | .475 |
| Income (b)[d] | -1.45 (0.98) | -3.37, 0.48 | .143 | 0.06 (0.09) | -0.11, 0.24 | .485 | 0.04 (0.08) | -0.12, 0.19 | .659 | 0.07 (0.07) | -0.07, 0.20 | .336 | -0.06 (0.12) | -0.30, 0.18 | .627 |
| ICC[e] | | .01 | | | <.01 | | | <.01 | | | .03 | | | <.01 | |

*Note*. Some of our model estimations resulted in a singularity error, and thus the random effects are unreliable. However, all models still converged. Intra-class correlations (ICCs) were low for these models (<5%), and for several of our dependent variables, the random effects intercept variance was 0. However, because group-level effects are of central theoretical interest to us, we decided to retain and report two levels in these models.

[a]The sample size across analyses ranged from *n* = 424 to *n* = 446.

[b]High school GPA was self-reported at baseline but would have been determined prior to the study (and thus before the other network items).

[c](w) represents within-group effects, which refers to the level of the individual (e.g., individuals associations within a learning community).

[d](b) represents between-group effects, which refers to the group mean level (e.g., differences between learning communities).

[e]Unadjusted intra-class correlations

*p < 0.05; **p < 0.01; ***p<0.001

## Appendix Table 4

*Cross-Sectional Associations Between Out-Eigenvector Centrality at the Individual and Group Level with Individuals' Adjustment (Full Covariates)*[a]

| Effect | High School GPA | | | Depression | | | Anxiety | | | Hostility | | | Institutional attachment | | |
|---|---|---|---|---|---|---|---|---|---|---|---|---|---|---|---|
| | *b* (SE) | *95% CI* | *p* | *b* (SE) | *95% CI* | *p* | *b* (SE) | *95% CI* | *p* | *b* (SE) | *95% CI* | *p* | *b* (SE) | *95% CI* | *p* |
| Intercept | **130.03 (28.13)** | **74.73, 185.32** | **<.001*****| 1.79 (2.44) | -3.01, 6.58 | .469 | 1.27 (2.17) | -3.00, 5.55 | .558 | 0.55 (2.02) | -3.41, 4.51 | .787 | **8.35 (3.43)** | **1.61, 15.09** | **.020*** |
| Out-Eigenvector Centrality (w)[c] | -1.39 (2.61) | -6.53, 3.75 | .596 | -0.23 (0.23) | -0.68, 0.23 | .330 | -0.31 (0.21) | -0.73, 0.10 | .136 | -0.24 (0.17) | -0.57, 0.09 | .149 | **0.84 (0.32)** | **0.21, 1.48** | **.009**** |
| Out- Eigenvector Centrality (b)[d] | -1.25 (12.96) | -26.72, 24.22 | .924 | -0.51 (1.11) | -2.70, 1.67 | .646 | 0.14 (0.99) | -1.81, 2.09 | .888 | 0.24 (0.90) | -1.54, 2.02 | .792 | 0.91 (1.56) | -2.15, 3.98 | .562 |
| Age (w)[c] | -0.19 (0.56) | -1.30, 0.92 | .741 | 0.06 (0.04) | -0.02, 0.14 | .133 | 0.05 (0.04) | -0.02, 0.12 | .187 | -0.03 (0.03) | -0.09, 0.02 | .250 | -0.06 (0.06) | -0.17, 0.05 | .305 |
| Age (b)[d] | -2.54 (1.45) | -5.39, 0.30 | .089 | 0.01 (0.13) | -0.24, 0.25 | .955 | 0.02 (0.11) | -0.20, 0.24 | .871 | 0.05 (0.10) | -0.15, 0.26 | .618 | -0.09 (0.18) | -0.44, 0.26 | .606 |
| Gender | 0.53 (0.87) | -1.18, 2.24 | .544 | 0.14 (0.08) | -0.02, 0.29 | .077 | **0.25 (0.07)** | **0.11, 0.38** | **.001***** | -0.01 (0.06) | -0.12, 0.10 | .850 | -0.02 (0.11) | -0.23, 0.20 | .875 |
| Learning Community Size | 0.11 (0.17) | -0.22, 0.44 | .522 | <0.01 (0.01) | -0.02, 0.03 | .762 | <.01 (0.01) | -0.03, 0.03 | .999 | -0.01 (0.01) | -0.03, 0.02 | .580 | 0.02 (0.02) | -0.02, 0.06 | .320 |
| Income (w)[c] | 0.25 (0.46) | -0.66, 1.16 | .595 | -0.05 (0.04) | -0.13, 0.04 | .280 | -0.04 (0.04) | -0.12, 0.04 | .294 | -0.05 (0.03) | -0.11, 0.01 | .089 | 0.04 (0.06) | -0.08, 0.15 | .541 |
| Income (b)[d] | -1.45 (1.00) | -3.42, 0.52 | .152 | 0.07 (0.09) | -0.10, 0.25 | .421 | 0.04 (0.08) | -0.12, 0.20 | .640 | 0.06 (0.07) | -0.07, 0.20 | .363 | -0.04 (0.13) | -0.29, 0.21 | .731 |
| ICC[e] | | .01 | | | <.01 | | | <.01 | | | .04 | | | .01 | |

*Note*. Some of our model estimations resulted in a singularity error, and thus the random effects are unreliable. However, all models still converged. Intra-class correlations (ICCs) were low for these models (<5%), and for several of our dependent variables, the random effects intercept variance was 0. However, because group-level effects are of central theoretical interest to us, we decided to retain and report two levels in these models.

[a]The sample size across analyses ranged from $n = 424$ to $n = 446$.

[b]High school GPA was self-reported at baseline but would have been determined prior to the study (and thus before the other network items).

[c](w) represents within-group effects, which refers to the level of the individual (e.g., individuals associations within a learning community).

[d](b) represents between-group effects, which refers to the group mean level (e.g., differences between learning communities).

[e]Unadjusted intra-class correlations

*p < 0.05; **p < 0.01; ***p<0.001

## Appendix Table 5

*Cross-Sectional Associations Between Reciprocated Friendship Ties at the Individual and Group Level with Individuals' Adjustment (Full Covariates)*[a]

| Effect | High School GPA | | | Depression | | | Anxiety | | | Hostility | | | Institutional attachment | | |
|---|---|---|---|---|---|---|---|---|---|---|---|---|---|---|---|
| | *b* (SE) | *95% CI* | *p* | *b* (SE) | *95% CI* | *p* | *b* (SE) | *95% CI* | *p* | *b* (SE) | *95% CI* | *p* | *b* (SE) | *95% CI* | *p* |
| Intercept | **130.28 (27.21)** | **76.79, 183.76** | **<.001*****  | 1.87 (2.36) | -2.76, 6.51 | .432 | 0.96 (2.11) | -3.18, 5.11 | .649 | 1.04 (1.95) | -2.80, 4.88 | .599 | **7.20 (3.22)** | **0.88, 13.52** | **.026*** |
| Reciprocated Friendship Ties (w)[c] | -0.18 (0.20) | -0.57, 0.21 | .359 | -0.03 (0.02) | -0.07, 0.00 | .066 | -0.03 (0.02) | -0.06, 0.00 | .055 | **-0.03 (0.01)** | **-0.06, -0.01** | **.009 **** | **0.09 (0.02)** | **0.04, 0.13** | **<.001***** |
| Reciprocated Friendship Ties (b)[d] | -0.02 (0.33) | -0.67, 0.63 | .951 | -0.02 (0.03) | -0.08, 0.04 | .465 | 0.03 (0.03) | -0.02, 0.08 | .196 | -0.02 (0.02) | -0.06, 0.03 | .505 | **0.10 (0.04)** | **0.02, 0.18** | **.013*** |
| Age (w)[c] | -0.20 (0.56) | -1.31, 0.91 | .721 | 0.06 (0.04) | -0.02, 0.14 | .136 | 0.05 (0.04) | -0.02, 0.12 | .171 | -0.03 (0.03) | -0.09, 0.02 | .235 | -0.06 (0.05) | -0.17, 0.05 | .294 |
| Age (b)[d] | -2.58 (1.45) | -5.44, 0.28 | .086 | -0.01 (0.13) | -0.25, 0.24 | .953 | 0.04 (0.11) | -0.18, 0.26 | .744 | 0.03 (0.10) | -0.17, 0.24 | .766 | -0.01 (0.17) | -0.35, 0.32 | .935 |
| Gender | 0.54 (0.87) | -1.17, 2.25 | .536 | 0.14 (0.08) | -0.01, 0.30 | .065 | **0.24 (0.07)** | **0.10, 0.38** | **.001 ***** | -0.01 (0.06) | -0.12, 0.10 | .888 | -0.03 (0.11) | -0.24, 0.18 | .786 |
| Learning Community Size | 0.12 (0.13) | -0.12, 0.37 | .328 | 0.01 (0.01) | -0.01, 0.04 | .206 | -0.01 (0.01) | -0.03, 0.01 | .406 | -0.01 (0.01) | -0.02, 0.01 | .519 | -0.01 (0.02) | -0.04, 0.02 | .539 |
| Income (w)[c] | 0.23 (0.46) | -0.68, 1.14 | .618 | -0.05 (0.04) | -0.13, 0.03 | .251 | -0.04 (0.04) | -0.12, 0.03 | .262 | -0.05 (0.03) | -0.11, <0.01 | .071 | 0.04 (0.06) | -0.07, 0.16 | .450 |
| Income (b)[d] | -1.47 (0.97) | -3.38, 0.43 | .132 | 0.07 (0.09) | -0.11, 0.24 | .453 | 0.03 (0.08) | -0.12, 0.19 | .659 | 0.07 (0.07) | -0.06, 0.21 | .300 | -0.04 (0.12) | -0.28, 0.20 | .736 |
| ICC[e] | | .01 | | | <.01 | | | <.01 | | | .04 | | | <.01 | |

*Note.* Some of our model estimations resulted in a singularity error, and thus the random effects are unreliable. However, all models still converged. Intra-class correlations (ICCs) were low for these models (<5%), and for several of our dependent variables, the random effects intercept variance was 0. However, because group-level effects are of central theoretical interest to us, we decided to retain and report two levels in these models.

[b]High school GPA was self-reported at baseline but would have been determined prior to the study (and thus before the other network items).

[c](w) represents within-group effects, which refers to the level of the individual (e.g., individuals associations within a learning community).

[d](b) represents between-group effects, which refers to the group mean level (e.g., differences between learning communities).

[e]Unadjusted intra-class correlations

*p < 0.05; **p < 0.01; ***p<0.001

## Appendix Table 6

*Longitudinal Associations Between Individual and Group Level In-Degree Centrality at Baseline and Individuals' Adjustment at Follow-up (Full Covariates)*[a]

| Effect | First-year GPA | | | Depression | | | Anxiety | | | Hostility | | | Institutional attachment | | |
|---|---|---|---|---|---|---|---|---|---|---|---|---|---|---|---|
| | *b* (SE) | *95% CI* | *p* | *b* (SE) | *95% CI* | *p* | *b* (SE) | *95% CI* | *p* | *b* (SE) | *95% CI* | *p* | *b* (SE) | *95% CI* | *p* |
| Intercept | 96.36 (48.84) | 0.30, 192.42 | .056 | 1.69 (2.83) | -3.87 (7.26) | .553 | 3.71 (2.39) | -1.00, 8.42 | .122 | 3.71 (2.12) | -0.46, 7.88 | .081 | 2.76 (3.93) | -4.97, 10.49 | .486 |
| In-degree Centrality (w)[b] | -0.25 (0.26) | *-0.76, 0.26* | .338 | 0.02 (0.02) | -0.02, 0.05 | .400 | 0.01 (0.02) | -0.02, 0.05 | .512 | 0.01 (0.02) | -0.02, 0.04 | .381 | -0.01 (0.02) | -0.05, 0.04 | .744 |
| In-degree Centrality (b)[c] | <0.01 (0.52) | *-1.02, 1.02* | .999 | <0.01 (0.03) | -0.07, 0.06 | .880 | 0.01 (0.03) | -0.04, 0.07 | .664 | -0.03 (0.02) | -0.08, 0.02 | .260 | 0.02 (0.04) | -0.06, 0.11 | .585 |
| Age (w)[b] | 0.30 (0.60) | -0.89, 1.48 | .620 | -0.03 (0.04) | -0.11, 0.06 | .524 | -0.01 (0.04) | -0.09, 0.06 | .706 | -0.05, 0.03 | -0.12, 0.02 | .166 | 0.01 (0.05) | -0.10, 0.11 | .892 |
| Age (b)[c] | -1.64 (2.60) | -6.76, 3.47 | .531 | -0.04 (0.15) | -0.33, 0.26 | .804 | -0.13 (0.13) | -0.38, 0.12 | .300 | -0.14, 0.11 | -0.36, 0.08 | .219 | 0.02 (0.19) | -0.36, 0.39 | .933 |
| Gender | **2.54 (1.24)** | **0.11, 4.97** | **.041*** | -0.08 (0.09) | -0.25, 0.09 | .339 | -0.05 (0.08) | -0.22, 0.11 | .502 | -0.03, 0.07 | -0.17, 0.11 | .678 | 0.13 (0.11) | -0.08, 0.34 | .224 |
| Learning Community Size | 0.12 (0.24) | -0.36, 0.59 | .629 | <0.01 (0.15) | -0.03, 0.03 | .959 | -0.01 (0.01) | -0.04, 0.01 | .319 | 0.01 (0.01) | -0.02, 0.03 | .609 | -0.01 (0.02) | -0.05, 0.03 | .689 |
| Income (w)[b] | -0.15 (0.58) | -1.30, 1.00 | .797 | -0.03 (0.20) | -0.42, 0.36 | .884 | -0.23 (0.19) | -0.60, 0.14 | .216 | -0.07, 0.16 | -0.39, 0.25 | .662 | -0.08 (0.25) | -0.57, 0.40 | .737 |
| Income (b)[c] | -0.76 (1.84) | -4.38, 2.86 | .682 | -0.02 (0.22) | -0.46, 0.42 | .926 | -0.19 (0.21) | -0.60, 0.22 | .368 | 0.08, 0.18 | -0.27, 0.44 | .643 | -0.15 (0.28) | -0.70, 0.40 | .584 |
| Credits Attempted (w)[b] | **0.72 (0.17)** | **0.38, 1.06** | **<.001 ***** | | | | | | | | | | | | |
| Credits Attempted (b)[c] | 0.12 (0.11) | *-0.09, 0.34* | .266 | | | | | | | | | | | | |
| Baseline Depression (w)[b] | | | | **0.64 (0.05)** | **0.53, 0.75** | **<.001***** | | | | | | | | | |
| Baseline Depression (b)[c] | | | | **0.57 (0.18)** | **0.21, 0.93** | **.003**** | | | | | | | | | |
| Baseline Anxiety (w)[b] | | | | | | | **0.56 (0.06)** | **0.45, 0.67** | **<.001***** | | | | | | |
| Baseline Anxiety (b)[c] | | | | | | | **0.40 (0.19)** | **0.02, 0.78** | **.038*** | | | | | | |
| Baseline Hostility (w)[b] | | | | | | | | | | **0.53 (0.06)** | **0.41, 0.66** | **<.001***** | | | |
| Baseline Hostility (b)[c] | | | | | | | | | | **0.34 (0.19)** | **-0.03, 0.70** | **.070** | | | |
| Baseline Institutional Attachment (w)[b] | | | | | | | | | | | | | **0.68 (0.05)** | **0.58, 078** | **<.001***** |
| Baseline Institutional Attachment (b)[c] | | | | | | | | | | | | | **0.54 (0.18)** | **0.18, 0.90** | **.005**** |
| ICC[d] | | .09 | | | .02 | | | <.01 | | | <.01 | | | .02 | |

*Note.* Some of our model estimations resulted in a singularity error, and thus the random effects are unreliable. However, all models still converged. Intra-class correlations (ICCs) were low for these models (<5%), and for several of our dependent variables, the random effects intercept variance was 0. However, because group-level effects are of central theoretical interest to us, we decided to retain and report two levels in these models.

[a]The sample size across analyses ranged from $n = 350$ to $n = 358$.

[b](w) represents within-group effects, which refers to the level of the individual (e.g., individuals associations within a learning community).

[c](b) represents between-group effects, which refers to the group mean level (e.g., differences between learning communities).

[d]Unadjusted intra-class correlations

*p < 0.05; **p < 0.01; ***p<0.001

## Appendix Table 7

*Longitudinal Associations Between Individual and Group Level Out-Degree Centrality at Baseline and Individuals' Adjustment at Follow-up (Full Covariates)*[a]

| Effect | First-year GPA | | | Depression | | | Anxiety | | | Hostility | | | Institutional attachment | | |
|---|---|---|---|---|---|---|---|---|---|---|---|---|---|---|---|
| | *b* (SE) | *95% CI* | *p* | *b* (SE) | *95% CI* | *p* | *b* (SE) | *95% CI* | *p* | *b* (SE) | *95% CI* | *p* | *b* (SE) | *95% CI* | *p* |
| Intercept | 95.80 (49.20) | -0.97, 192.58 | .059 | 1.76 (2.83) | -3.81, 7.34 | .538 | 3.76 (2.39) | -0.95, 8.47 | .118 | 3.76 (2.12) | -0.42, 7.93 | .078 | 2.79 (3.93) | -4.94, 10.52 | .482 |
| Out-degree Centrality (w)[b] | -0.24 (0.21) | -0.65, 0.17 | .246 | 0.01 (0.01) | -0.02, 0.04 | .570 | <0.01 (0.01) | -0.02, 0.03 | .739 | <0.01 (0.01) | -0.02, 0.03 | .786 | 0.02 (0.02) | -0.02, 0.05 | .389 |
| Out-degree Centrality (b)[c] | -0.02 (0.52) | -1.04, 1.01 | .977 | -0.01, (0.03) | -0.07, 0.06 | .876 | 0.01 (0.03) | -0.04, 0.07 | .665 | -0.03 (0.02) | -0.08. 0.02 | .262 | 0.02 (0.04) | -0.06, 0.11 | .615 |
| Age (w)[b] | 0.25 (0.60) | -0.94, 1.43 | .683 | -0.03 (0.04) | -0.11, 0.06 | .527 | -0.01 (0.04) | -0.09, 0.06 | .707 | -0.05 (0.03) | -0.12 0.02 | .163 | 0.01 (0.05) | -0.09, 0.11 | .850 |
| Age (b)[c] | -1.62 (2.62) | -6.78, 3.54 | .541 | -0.04 (0.15) | -0.34, 0.26 | .790 | -0.02 (0.13) | -0.39, 0.12 | .295 | -0.14 (0.11) | -0.36, 0.08 | .214 | 0.01 (0.19) | -0.36, 0.39 | .945 |
| Gender | **2.73 (1.25)** | **0.28, 5.18** | **.029*** | -0.09 (0.09) | -0.26, 0.08 | .308 | -0.06 (0.08) | -0.22, 0.10 | | -0.03 (0.07) | -0.17, 0.11 | .647 | 0.12 (0.11) | -0.09, 0.34 | .255 |
| Learning Community Size | 0.12 (0.24) | -0.36, 0.60 | .616 | <0.01, - 0.02) | -0.03, 0.03 | .976 | -0.01 (0.01) | -0.04, 0.01 | .476 | 0.01 (0.01) | -0.02, 0.03 | .626 | -0.01 (0.02) | -0.05, 0.03 | .682 |
| Income (w)[b] | -0.13 (0.58) | -1.27, 1.02 | .830 | -0.03 (0.20) | -0.42, 0.37 | .890 | -0.23 (0.19) | -0.60, 0.14 | .216 | -0.07 (0.16) | -0.39, 0.25 | .659 | -0.08 (0.25) | -0.57, 0.41 | .745 |
| Income (b)[c] | -0.75 (1.85) | -4.40, 2.89 | .685 | -0.02 (0.22) | -0.46, 0.42 | .931 | -0.19 (0.21) | -0.60, 0.22 | .370 | 0.08 (0.18) | -0.27, 0.44 | .641 | -0.16 (0.28) | -0.70, 0.39 | .578 |
| Credits Attempted (w)[b] | **0.72 (0.17)** | **0.38, 1.06** | **<.001*** | | | | | | | | | | | | |
| Credits Attempted (b)[c] | 0.12 (0.11) | -0.09, 0.34 | .272 | | | | | | | | | | | | |
| Baseline Depression (w)[b] | | | | **0.64 (0.06)** | **0.54, 0.75** | **<.001*** | | | | | | | | | |
| Baseline Depression (b)[c] | | | | **0.57 (0.19)** | **0.20, 0.93** | **.004** | | | | | | | | | |
| Baseline Anxiety (w)[b] | | | | | | | **0.56 (0.06)** | **0.45, 0.67** | **<.001*** | | | | | | |
| Baseline Anxiety (b)[c] | | | | | | | **0.40 (0.19)** | **0.02, 0.78** | **.041*** | | | | | | |
| Baseline Hostility (w)[b] | | | | | | | | | | **0.53 (0.06)** | **0.41, 0.66** | **<.001*** | | | |
| Baseline Hostility (b)[c] | | | | | | | | | | .33 (0.19) | -0.03, 0.70 | .075 | | | |
| Baseline Institutional Attachment (w)[b] | | | | | | | | | | <.01 | | | **0.67 (0.05)** | **0.57, 0.66** | **<.001*** |
| Baseline Institutional Attachment (b)[c] | | | | | | | | | | | | | **0.54 (0.18)** | **0.18, 0.90** | **0.005*** |
| ICC[d] | | .09 | | | .02 | | | <.01 | | | <.01 | | | .02 | |

*Note.* Some of our model estimations resulted in a singularity error, and thus the random effects are unreliable. However, all models still converged. Intra-class correlations (ICCs) were low for these models (<5%), and for several of our dependent variables, the random effects intercept variance was 0. However, because group-level effects are of central theoretical interest to us, we decided to retain and report two levels in these models.

[a]The sample size across analyses ranged from *n* = 350 to *n* = 358.

[b](w) represents within-group effects, which refers to the level of the individual (e.g., individuals associations within a learning community).

[c](b) represents between-group effects, which refers to the group mean level (e.g., differences between learning communities).

[d]Unadjusted intra-class correlations

*p < 0.05; **p < 0.01; ***p<0.001

## Appendix Table 8

*Longitudinal Associations Between Individual and Group Level In-Eigenvector Centrality at Baseline and Individuals' Adjustment at Follow-up (Full Covariates)*[a]

| Effect | First-year GPA[b] | | | Depression | | | Anxiety | | | Hostility | | | Institutional attachment | | |
|---|---|---|---|---|---|---|---|---|---|---|---|---|---|---|---|
| | *b* (SE) | *95% CI* | *p* | *b* (SE) | *95% CI* | *p* | *b* (SE) | *95% CI* | *p* | *b* (SE) | *95% CI* | *p* | *b* (SE) | *95% CI* | *p* |
| Intercept | 96.71 (44.99) | 8.21, 185.22 | .038 | 1.73 (2.79) | -3.76, 7.23 | .539 | 4.09 (2.34) | -0.52, 8.70 | .082 | 3.43 (2.07) | -0.64, 7.50 | .098 | 3.09 (3.71) | -4.21, 10.39 | .410 |
| In-Eigenvector Centrality (w)[c] | -3.60 (3.98) | -11.43, 4.22 | .366 | 0.09 (0.28) | -0.47, 0.65 | .756 | 0.37 (0.27) | -0.15, 0.90 | .162 | -0.10 (0.23) | -0.55, 0.36 | .678 | -0.36 (0.35) | -1.05, 0.34 | .313 |
| In-Eigenvector Centrality (b)[d] | **23.85 (8.90)** | **6.35, 41.35** | **.009**** | -0.37 (0.78) | -1.71, 0.96 | .584 | -0.02 (0.60) | -1.41, 0.95 | .702 | -1.03 (0.52) | -2.07, <0.01 | .049 | **1.87 (0.83)** | **0.24, 3.50** | **.026*** |
| Age (w)[c] | 0.25 (0.60) | -0.93, 1.43 | .676 | -0.03 (0.04) | -0.11, 0.06 | .517 | -0.02 (0.04) | -0.09, 0.06 | .683 | -0.05 (0.03) | -0.11, 0.02 | .173 | 0.01 (0.05) | -0.10, 0.11 | .907 |
| Age (b)[d] | -2.07 (2.40) | -6.79, 2.66 | .395 | -0.03 (0.15) | -0.33, 0.26 | .823 | -0.15 (0.13) | -0.40, 0.10 | .231 | -0.11 (0.11) | -0.32, 0.11 | .330 | -0.02 (0.18) | -0.37, 0.33 | .901 |
| Gender | 2.29 (1.22) | -0.12, 4.69 | .063 | -0.08 (0.09) | -0.25, 0.09 | .339 | -0.06 (0.08) | -0.22, 0.10 | .493 | -0.03 (0.07) | -0.17, 0.11 | .656 | 0.13 (0.11) | -0.08, 0.34 | .237 |
| Learning Community Size | 0.29 (0.19) | -0.08, 0.66 | .127 | <0.01 (0.01) | -0.03, 0.02 | .750 | -0.01 (0.01) | -0.03, 0.01 | .292 | -0.01 (0.01) | -0.03, 0.01 | .262 | 0.02 (0.02) | -0.01, 0.05 | .316 |
| Income (w)[c] | -0.16 (0.58) | -1.30, 0.99 | .788 | -0.03 (0.20) | -0.43, 0.36 | .869 | -0.25 (0.19) | -0.62, 0.21 | .181 | -0.07 (0.16) | -0.39, 0.26 | .689 | -0.08 (0.25) | -0.56, 0.41 | .761 |
| Income (b)[d] | -1.28 (1.76) | -4.74, 2.17 | .469 | -0.02 (0.23) | -0.46, 0.43 | .941 | -0.21 (0.21) | -0.62, 0.21 | .327 | 0.11 (0.18) | -0.25, 0.47 | .535 | -0.18 (0.28) | -0.73, 0.37 | .514 |
| Credits Attempted (w)[c] | **0.72 (0.17)** | **0.38, 1.06** | **<0.001***** | | | | | | | | | | | | |
| Credits Attempted (b)[d] | 0.11 (0.10) | -0.09. 0.31 | 0.277 | | | | | | | | | | | | |
| Baseline Depression (w)[c] | | | | **0.64 (0.05)** | **0.53, 0.75** | **<.001***** | | | | | | | | | |
| Baseline Depression (b)[d] | | | | **0.58 (0.19)** | **0.21, 0.95** | **.003**** | | | | | | | | | |
| Baseline Anxiety (w)[c] | | | | | | | **0.56 (0.19)** | **0.45, 0.67** | **<.001***** | | | | | | |
| Baseline Anxiety (b)[d] | | | | | | | **0.42 (0.19)** | **0.04, 0.80** | **.030*** | | | | | | |
| Baseline Hostility (w)[c] | | | | | | | | | | **0.53 (0.06)** | **0.40, 0.65** | **<.001***** | | | |
| Baseline Hostility (b)[d] | | | | | | | | | | **-0.37 (0.19)** | **0.01, 0.74** | **.046*** | | | |
| Baseline Institutional Attachment (w)[c] | | | | | | | | | | | | | **0.68 (0.05)** | **0.58, 0.78** | **<.001***** |
| Baseline Institutional Attachment (b)[d] | | | | | | | | | | | | | **0.50 (0.17)** | **0.17. 0.83** | **.005**** |
| ICC[e] | | .07 | | | .02 | | | <.01 | | | <.01 | | | .01 | |

*Note*. Some of our model estimations resulted in a singularity error, and thus the random effects are unreliable. However, all models still converged. Intra-class correlations (ICCs) were low for these models (<5%), and for several of our dependent variables, the random effects intercept variance was 0. However, because group-level effects are of central theoretical interest to us, we decided to retain and report two levels in these models.

[a]The sample size across analyses ranged from $n = 350$ to $n = 358$.

[b]The association between in-eigenvector centrality and first-year GPA should be interpreted with caution due to small group sizes. As in-eigenvector centrality is a weighted score based on how well-connected one's connections are, it is particularly sensitive to disparities in group sizes.

[c](w) represents within-group effects, which refers to the level of the individual (e.g., individuals associations within a learning community).

[d](b) represents between-group effects, which refers to the group mean level (e.g., differences between learning communities).

[e]Unadjusted intra-class correlations

*p < 0.05; **p < 0.01; ***p<0.001

## Appendix Table 9

*Longitudinal Associations Between Individual and Group Level Out-Eigenvector Centrality at Baseline and Individuals' Adjustment at Follow-up (Full Covariates)[a]*

| Effect | First-year GPA | | | Depression | | | Anxiety | | | Hostility | | | Institutional attachment | | |
|---|---|---|---|---|---|---|---|---|---|---|---|---|---|---|---|
| | *b* (SE) | *95% CI* | *p* | *b* (SE) | *95% CI* | *p* | *b* (SE) | *95% CI* | *p* | *b* (SE) | *95% CI* | *p* | *b* (SE) | *95% CI* | *p* |
| Intercept | **108.42 (50.03)** | **10.02 (206.82)** | **.036*** | 0.82 (2.80) | -4.69, 6.34 | .771 | 3.63 (2.42) | -1.13, 8.39 | .134 | 3.03 (2.14) | -1.19, 7.24 | .159 | 1.71 (3.93) | -6.03, 9.45 | .667 |
| Out-Eigenvector Centrality (w)[b] | -3.26 (3.57) | -10.28, 3.75 | .361 | 0.22 (0.25) | -0.28, 0.72 | .381 | **0.52 (0.24)** | **0.05, 0.99** | **.029*** | 0.10 (0.21) | -0.32, 0.51 | .650 | -0.25 (0.32) | -0.88, 0.37 | .429 |
| Out-Eigenvector Centrality (b)[c] | -18.01 (21.51) | -60.33, 24.30 | .406 | 1.57 (1.26) | -0.91, 4.04 | .222 | 0.89 (1.09) | -1.25, 3.04 | .414 | 0.46 (0.97) | -1.45, 2.36 | .637 | 1.90 (1.59) | -1.22, 5.02 | .240 |
| Age (w)[b] | 0.24 (0.60) | -0.95, 1.43 | .690 | -0.02 (0.04) | -0.11, 0.06 | .567 | -0.01 (0.04) | -0.09, 0.07 | .812 | -0.05 (0.03) | -0.11, 0.02 | .189 | 0.01 (0.05) | -0.10, 0.11 | .911 |
| Age (b)[c] | -1.92 (2.57) | -6.99, 3.14 | .459 | -0.02 (0.14) | -0.31, 0.26 | .887 | -0.14 (0.13) | -0.39, 0.10 | .252 | -0.11 (0.11) | -0.33, 0.11 | .323 | 0.02 (0.18) | -0.34, 0.39 | .893 |
| Gender | **2.55 (1.24)** | **0.12, 4.99** | **.040*** | -0.09 (0.09) | -0.26, 0.08 | .294 | -0.07 (0.08) | -0.23, 0.09 | .395 | -0.03 (0.07) | -0.17, 0.11 | .630 | 0.14 (0.11) | -0.07. 0.36 | .183 |
| Learning Community Size | -0.07 (0.30) | -0.65, 0.51 | .813 | 0.02 (0.02) | -0.02, 0.05 | .385 | <0.01 (0.01) | -0.03, 0.03 | .933 | <0.01 (0.01) | -0.02, 0.03 | .899 | 0.02 (0.02) | -0.02, 0.06 | .389 |
| Income (w)[b] | -0.11 (0.58) | -1.26, 1.03 | .840 | -0.02 (0.20) | -0.42, 0.37 | .909 | -0.24 (0.19) | -0.61, 0.12 | .195 | -0.07 (0.18) | -0.40, 0.25 | .660 | -0.05 (0.25) | -0.54, 0.44 | .844 |
| Income (b)[c] | -0.39 )1.87) | -4.08, 3.30 | .836 | -0.05 (0.02) | -0.49, 0.39 | .834 | -0.22 (0.21) | -0.63, 0.19 | .291 | 0.07 (0.18) | -0.29, 0.43 | .692 | -0.15 (0.28) | -0.70, 0.40 | .588 |
| Credits Attempted (w)[b] | **0.72 (0.17)** | **0.38, 1.06** | **<.001*****| | | | | | | | | | | | |
| Credits Attempted (b)[c] | 0.12 (0.11) | -0.09, 0.33 | 0.270 | | | | | | | | | | | | |
| Baseline Depression (w)[b] | | | | **0.65 (0.06)** | **0.54, 0.75** | **<.001***** | | | | | | | | | |
| Baseline Depression (b)[c] | | | | **0.56 (0.18)** | **0.20, 0.92** | **.003**** | | | | | | | | | |
| Baseline Anxiety (w)[b] | | | | | | | **0.57 (0.06)** | **0.46, 0.68** | **<.001***** | | | | | | |
| Baseline Anxiety (b)[c] | | | | | | | **0.39 (0.19)** | **0.01, 0.77** | **.042*** | | | | | | |
| Baseline Hostility (w)[b] | | | | | | | | | | **0.53 (0.06)** | **0.40, 0.65** | **<.001***** | | | |
| Baseline Hostility (b)[c] | | | | | | | | | | 0.33 (0.19) | -0.04, 0.70 | 0.080 | | | |
| Baseline Institutional Attachment (w)[b] | | | | | | | | | | | | | **0.68 (0.05)** | **0.58, 0.78** | **<.001***** |
| Baseline Institutional Attachment (b)[c] | | | | | | | | | | | | | **0.56 (0.17)** | **0.23, 0.90** | **.002**** |
| ICC[d] | | .09 | | | .02 | | | <.01 | | | <.01 | | | .02 | |

*Note.* Some of our model estimations resulted in a singularity error, and thus the random effects are unreliable. However, all models still converged. Intra-class correlations (ICCs) were low for these models (<5%), and for several of our dependent variables, the random effects intercept variance was 0. However, because group-level effects are of central theoretical interest to us, we decided to retain and report two levels in these models.

[a]The sample size across analyses ranged from *n* = 350 to *n* = 358.

[b](w) represents within-group effects, which refers to the level of the individual (e.g., individuals associations within a learning community).

[c](b) represents between-group effects, which refers to the group mean level (e.g., differences between learning communities).

[d]Unadjusted intra-class correlations

*p < 0.05; **p < 0.01; ***p<0.001

## Appendix Table 10

*Longitudinal Associations Between Individual and Group Level Reciprocated Friendship Ties at Baseline and Individuals' Adjustment at Follow-up (Full Covariates)*[a]

| Effect | First-year GPA | | | Depression | | | Anxiety | | | Hostility | | | Institutional attachment | | |
|---|---|---|---|---|---|---|---|---|---|---|---|---|---|---|---|
| | *b* (SE) | *95% CI* | *p* | *b* (SE) | *95% CI* | *p* | *b* (SE) | *95% CI* | *p* | *b* (SE) | *95% CI* | *p* | *b* (SE) | *95% CI* | *p* |
| Intercept | 92.96 (48.84) | -3.10, 189.01 | .065 | 1.66 (2.83) | -3.90, 7.21 | .562 | 3.89 (2.40) | -0.84, 8.61 | .107 | 3.81 (2.13) | -0.39, 8.01 | .075 | 2.75 (3.94) | -5.00, 10.49 | .490 |
| Reciprocated Friendship Ties (w)[b] | -0.24 (0.27) | -0.77, 0.30 | .387 | 0.02 (0.02) | -0.01, 0.06 | .195 | 0.01 (0.02) | -0.02, 0.05 | .425 | 0.01 (0.02) | -0.02, 0.04 | .596 | 0.01 (0.02) | -0.04, 0.05 | .834 |
| Reciprocated Friendship Ties (b)[c] | 0.23 (0.56) | -0.88, 1.34 | .683 | <0.01 (0.04) | -0.07, 0.07 | .909 | <0.01 (0.03) | -0.06. 0.06 | .966 | -0.03 (0.03) | -0.08, 0.02 | .246 | 0.01 (0.05) | -0.08, 0.10 | .782 |
| Age (w)[b] | 0.27 (0.60) | -0.91. 1.46 | .649 | -0.03 (0.04) | -0.11, 0.06 | .545 | -0.01 (0.04) | -0.09, 0.06 | .707 | -0.05 (0.03) | -0.12, 0.02 | .168 | 0.01 (0.05) | -0.09, 0.11 | .889 |
| Age (b)[c] | -1.47 (2.60) | -6.59, 3.65 | .576 | -0.03 (0.15) | -0.33, 0.26 | .820 | -0.14 (0.13) | -0.39, 0.11 | .269 | -0.14 (0.11) | -0.36, 0.08 | .211 | 0.01 (0.19) | -0.37, 0.39 | .954 |
| Gender | **2.60 (1.24)** | **0.16, 5.04** | **.037*** | -0.09 (0.09) | -0.26, 0.08 | .299 | -0.06 (0.08) | -0.22, 0.10 | .466 | -0.03 (0.07) | -0.17, 0.11 | .654 | 0.13 (0.11) | -0.08, 0.34 | .222 |
| Learning Community Size | 0.07 (0.22) | -0.36, 0.50 | .741 | <0.01 (0.01) | -0.03, 0.03 | .972 | -0.01 (0.01) | -0.03, 0.01 | .398 | <0.01 (0.01) | -0.02, 0.02 | .741 | <0.01 (0.02) | -0.04, 0.03 | .838 |
| Income (w)[b] | -0.14 (0.58) | -1.29, 1.01 | .808 | -0.03 (0.20) | -0.42, 0.36 | .886 | -0.23 (0.19) | -0.60, 0.14 | .217 | -0.07 (0.16) | -0.39, 0.25 | .676 | -0.08 (0.25) | -0.57, 0.40 | .737 |
| Income (b)[c] | -0.78 (1.84) | -4.39, 2.84 | .675 | -0.02 (0.22) | -0.46, 0.42 | .921 | -0.19 (0.21) | -0.60, 0.22 | .371 | 0.09 (0.18) | -0.27, 0.45 | .620 | -0.16 (0.28) | -0.71, .40 | .579 |
| Credits Attempted (w)[b] | **0.72 (0.17)** | **0.38, 1.06** | **<.001***** | | | | | | | | | | | | |
| Credits Attempted (b)[c] | 0.12 (0.11) | -0.09, 0.33 | .273 | | | | | | | | | | | | |
| Baseline Depression (w)[b] | | | | **0.65 (0.06)** | **0.54, 0.75** | **<.001***** | | | | | | | | | |
| Baseline Depression (b)[c] | | | | **0.57 (0.18)** | **0.20, 0.93** | **.003**** | | | | | | | | | |
| Baseline Anxiety (w)[b] | | | | | | | **0.57 (0.06)** | **0.46, 0.68** | **<.001***** | | | | | | |
| Baseline Anxiety (b)[c] | | | | | | | **0.41 (0.19)** | **0.03, 0.79** | **0.034*** | | | | | | |
| Baseline Hostility (w)[b] | | | | | | | | | | **0.53 (0.06)** | **0.41, 0.66** | **<.001***** | | | |
| Baseline Hostility (b)[c] | | | | | | | | | | 0.32 (0.19) | -0.05, 0.68 | .092 | | | |
| Baseline Institutional Attachment (w)[b] | | | | | | | | | | | | | **0.68 (0.05)** | **0.58, 0.78** | **<.001***** |
| Baseline Institutional Attachment (b)[c] | | | | | | | | | | | | | **0.55 (0.18)** | **0.19, 0.91** | **.004**** |
| ICC[d] | | .09 | | | .02 | | | <.01 | | | <.01 | | | .02 | |

*Note.* Some of our model estimations resulted in a singularity error, and thus the random effects are unreliable. However, all models still converged. Intra-class correlations (ICCs) were low for these models (<5%), and for several of our dependent variables, the random effects intercept variance was 0. However, because group-level effects are of central theoretical interest to us, we decided to retain and report two levels in these models.

[a]The sample size across analyses ranged from $n = 350$ to $n = 358$.

[b](w) represents within-group effects, which refers to the level of the individual (e.g., individuals associations within a learning community).

[c](b) represents between-group effects, which refers to the group mean level (e.g., differences between learning communities).

[d]Unadjusted intra-class correlations

*p < 0.05; **p < 0.01; ***p<0.001
